# Lattice excitations in NdFeO_3_ through polarized optical spectroscopies

**DOI:** 10.1038/s41598-024-66594-w

**Published:** 2024-07-04

**Authors:** M. M. Gomes, R. Vilarinho, H. Zhao, J. Íñiguez-González, M. Mihalik, M. Mihalik, A. Maia, V. Goian, D. Nuzhnyy, S. Kamba, J. Agostinho Moreira

**Affiliations:** 1 IFIMUP, LaPMET, Departamento de Física e Astronomia da Faculdade de Ciências, Universidade do Porto, Rua do Campo Alegre S/N, 4169-007 Porto, Portugal; 2 Key Laboratory of Material Simulation Methods and Software of Ministry of Education, College of Physics, Jilin University, Changchun, 130012 China; 3 Materials Research and Technology Department, Luxembourg Institute of Science and Technology, 5 Avenue des Hauts-Fourneaux, 4362 Esch/Alzette, Luxembourg; 4 Department of Physics and Materials Science, University of Luxembourg, 41 Rue du Brill, 4422 Belvaux, Luxembourg; 5 Institute of Experimental Physics, Slovak Academy of Sciences, Watsonova 47, 040 01 Košice, Slovak Republic; 6 Institute of Physics of the Czech Academy of Sciences, Na Slovance 2, 182 00 Prague 8, Czech Republic

**Keywords:** Physics, Condensed-matter physics

## Abstract

The possibility of inducing new polar and/or magnetic transient states through the pumping of optical phonons towards the non-linear regime has renewed the scientific interest in orthoferrites. Nonetheless, to perform these studies it is fundamental to have a deep knowledge of the lattice excitations at equilibrium conditions. In this work, we present a complete characterization of the optically-active zone-center phonons in NdFeO_3_ single crystals at room temperature by means of polarized Raman and infrared spectroscopies. The study is complemented with polarized infrared spectroscopy at 4 K and unpolarized Raman scattering at 10 K. The predicted polar phonons were successfully observed together with some of the crystal-field excitations. First-principles simulations further allow the eigenmode and symmetry assignments of the optical phonons. The calculated atomic motions of each mode are of significant interest, as they are common for all orthoferrites and to most of the large family of orthorhombic *Pbnm* perovskites.

## Introduction

NdFeO_3_ has been recently the focus of intense research because it opens the opportunity to induce non-equilibrium transient magnetic states by driving optical phonons towards the non-linear regime, through the use of intense and ultrashort THz pulses, as it has been done for other orthoferrites^1–4^. In particular, the manipulation of magnetic domains and photoinduced magnetic transitions between equilibrium and nonequilibrium states has been possible due to the excitation of polar phonons^1–4^. NdFeO_3_ is an interesting candidate for these types of studies due to its magnetic properties and the interplay between the Nd^3+^ and Fe^3+^ magnetic sublattices. The coupling between magnetism and structure has been addressed in the recent past by temperature dependent unpolarized Raman scattering experiments in NdFeO_3_ ceramic samples in a broad temperature range^5^. Nonetheless, a deep knowledge of the zone-center lattice excitations is required and for the case of NdFeO_3_ only the optical Raman-active phonons and spin waves excitations have been characterized^6,7^. The infrared (IR)-active phonons in this compound have yet to be systematically studied, as the few publications available only present a rather incomplete characterization^8,9^.

To fill this gap, this work presents a full characterization of the optically-active zone center lattice excitations in oriented NdFeO_3_ single crystals, by means of polarized IR and Raman spectroscopies at 300 K and 4 K. To complement the experimental results, first-principles calculations were performed to confirm the mode symmetry and identify the vibrational motion of each phonon. Such motions are valid for a large number of materials sharing the *Pbnm* perovskite structure.

## NdFeO_3_ phase sequence: a short overview

At room temperature, NdFeO_3_ exhibits an orthorhombically distorted perovskite structure with *Pbnm* space group^6^. NdFeO_3_ undergoes a paramagnetic to antiferromagnetic phase transition at T_N_ = 760 K^10^. Below T_N_, the Fe^3+^ magnetic momenta order in the G_x_A_y_F_z_ configuration (for details see e.g. Ref.^4^), triggered by the condensation of the magnetic order parameter with symmetry Γ_4_, giving rise to a net magnetization that appears along the *c*-axis. On further cooling, the competition of the Fe–Fe and Nd–Fe interactions leads to a spin-reorientation transition (SRT) of the ordered Fe^3+^ magnetic momenta, triggered by the condensation of another order parameter with symmetry Γ_2_, in the 170–100 K temperature range^10,11^. In this temperature interval, the net magnetization continuously rotates from the *c*- to the *a*-axis, and although several authors have assumed the *Pbnm* space group in this region^12,13^, the mixed-phase symmetry Γ_4_–Γ_2_ is not compatible with the orthorhombic symmetry. Therefore, during the SRT, the system should adopt a monoclinic structure^14^. The SRT has been correlated with the coherent rotation of FeO_6_ octahedra accompanied by an increase in the Fe–O–Fe angle with increasing temperature^12^. More recently, a magnetostructural effect was discovered in NdFeO_3_, where, at the limits of the SRT, a deviation from the expected temperature dependence of a Raman-active FeO_6_ rotational mode is observed^5^. Moreover, the Nd^3+^ oscillation mode evidences a cross-talk of the Fe^3+^/Nd^3+^ spins through magnetic interactions between the two magnetic sublattices. At the end of the spin-reorientation process (below 100 K), the Fe^3+^ ordering is described by the F_x_C_y_G_z_ structure (symmetry Γ_2_), with a net magnetization along the *a*-axis, and the crystal structure is once again described by the *Pbnm* space group. Hereafter, the magnetization decreases due to the gradual antiparallel ordering of the Nd^3+^ magnetic momenta in the exchange field of the Fe^3+^. At 7.6 K, the magnetic contributions of the Nd^3+^ and Fe^3+^ spins compensate and the magnetization reverses for lower temperatures^15^. The ordering transition of the Nd^3+^ spins only occurs around 1.5 K^16^.

## Experimental

NdFeO_3_ single crystals were grown in an optical-floating zone furnace, as described elsewhere^17^. Three single domain platelets were oriented along the three orthorhombic directions using Laue patterns and polished.

Low-temperature IR reflectivity measurements in the 50–650 cm^−1^ frequency range were performed using a Bruker IFS-113v Fourier-transform IR spectrometer. The detector is a Si bolometer, cooled to 1.6 K using liquid He. For the spectral region 5–50 cm^−1^ a custom-made time-domain terahertz (THz) spectrometer measuring both the transmission signal and its phase was used. This allowed to directly calculate the complex permittivity and subsequently the reflectivity below 50 cm^−1^. The sample temperature was controlled by Oxford Instruments Optistat optical continuous He-flow cryostats, with polyethylene and mylar windows used for IR and THz measurements, respectively. The IR spectra were fitted using a generalized-oscillator model with the factorized form of the complex permittivity:

1 $$\epsilon \left(\omega \right)={\epsilon }{\prime}\left(\omega \right)+i{\epsilon }{\prime}{\prime}\left(\omega \right)={\epsilon }_{\infty }\prod_{j}\frac{{\omega }_{LOj}^{2}-{\omega }^{2}+i\omega {\gamma }_{LOj}}{{\omega }_{TOj}^{2}-{\omega }^{2}+i\omega {\gamma }_{TOj}}$$

where ${\omega }_{TOj}$ and ${\omega }_{LOj}$ stand for transverse and longitudinal frequencies of the *j*-th polar phonon, respectively, and ${\gamma }_{TOj}$ and ${\gamma }_{LOj}$ are the corresponding damping constants^18^. The high-frequency permittivity, ${\epsilon }_{\infty }$, originating from electronic absorption processes, was obtained from the room-temperature frequency-independent reflectivity tail above the phonon frequencies and was assumed temperature independent. The complex permittivity, $\epsilon (\omega )$, is related to the reflectivity, $R(\omega )$, by:

2 $$R(\omega )= {\left|\frac{\sqrt{\epsilon (\omega )}-1}{\sqrt{\epsilon (\omega )}+1}\right|}^{2}$$

The polarized Raman spectra were recorded using a Renishaw in Via Qontor spectrometer with a 532 nm linearly polarized diode-pumped laser, in the spectral range 100–800 cm^−1^. The effect of the laser power on the Raman spectra was studied previously to prevent the heating of the sample. The unpolarized Raman scattering data at 10 K were taken from Ref.^5^, using the same spectrometer and laser excitation, with the sample placed in an APD closed-cycle He cryostat. A sum of damped harmonic oscillators was fitted to the experimental spectra to obtain the modes wavenumber and linewidth^19^:

3 $$I\left(\omega ,T\right)={\left[1+n\left(\omega ,T\right)\right]}^{-1}\sum_{j}\frac{\omega {A}_{0j}{\Omega }_{0j}^{2}{\Gamma }_{0j}}{{\left({\Omega }_{0j}^{2}-{\omega }^{2}\right)}^{2}+{\omega }^{2}{\Gamma }_{0j}^{2}}$$

where $n(\omega ,T)$ is the Bose–Einstein factor and ${A}_{0j}, {\Omega }_{0j}, {\Gamma }_{0j}$ are, respectively, the strength, wavenumber, and damping coefficient of the *j*-th oscillator.

Density functional theory as implemented in the VASP software^20–22^ was employed for the first-principles calculations. We used the projector augmented wave (PAW) approximation^23^ to treat the ionic cores, considering only the following electrons explicitly in the calculations: O’s 2*s* and 2*p*; Fe’s 3*s*, 3*p*, 3*d*, and 3*s*; and Nd’s 5*s*, 5*p*, 6*s* and, additionally, 1 of the 4*f* electrons. (As frequently done, Nd was treated as a 3 + cation, with a nominal configuration of 4*f*^2^5*d*^0^5*s*^0^ and 2 4*f* electrons included the ionic core.) We used the generalized gradient approximation to treat the exchange–correlation potential, in particular the so-called “PBEsol” scheme^24^. The kinetic energy cutoff was set to 550 eV, and the k-point mesh of 6 × 6 × 4 (for the 20-atom cell in the *Pbnm* setting) was used for sampling the Brillouin zone. The ionic force threshold for structural relaxations was set to 0.005 eV/Å. An effective Hubbard *U* of 4.0 eV (using the approach introduced by Dudarev et al.^25^) was chosen to better treat the correlation effect of Fe’s 3*d* electrons. As for Nd ion, the 4*f* electrons kept within the core do not require any Hubbard *U* correction.

## Results

### Group theory analysis of the phonons

In the temperature range where the *Pbnm* space group describes the crystallographic structure of NdFeO_3_, Fe atoms occupy the B-sites of a perovskite-type structure at the octahedron centers and they are surrounded by a network of corner-sharing oxygen octahedra, while the Nd atoms occupy the A-sites at the corners of the cube in the perovskite-type structure. Due to the octahedra tilting in the *a*^−^*a*^−^*c*^+^ scheme (Glazer notation)^26^, the oxygen polyhedra are distorted and two inequivalent oxygen positions can be defined: the apical O1 and the equatorial O2. In the Wyckoff notation, the crystallographic positions are: Nd in 4c, Fe in 4b, O1 in 4c and O2 in 8d. Group theoretical analysis gives the following decomposition into irreducible representations of the factor group:

4 $$\Gamma_{phonons} = 7A_{g} + 7B_{1g} + 5B_{2g} + 5B_{3g} + 8A_{u} + 8B_{1u} + 10B_{2u} + 10B_{3u}$$

All zone-center phonons are nondegenerate, and the 8 A_u_ phonons are optically silent. The optical phonons transforming according to an even irreducible representations are Raman-active (Γ_Raman_ = 7A_g_ + 7B_1g_ + 5B_2g_ + 5B_3g_), while those transforming according to the B-odd representations are IR-active (Γ_IR_ = 7B_1u_ + 9B_2u_ + 9B_3u_). Three phonons (1B_1u_ + 1B_2u_ + 1B_3u_) are acoustic phonons. As the Fe sits in the center of inversion, its motions only participate in the polar phonons.

### Raman-active modes

**Figure 1:**
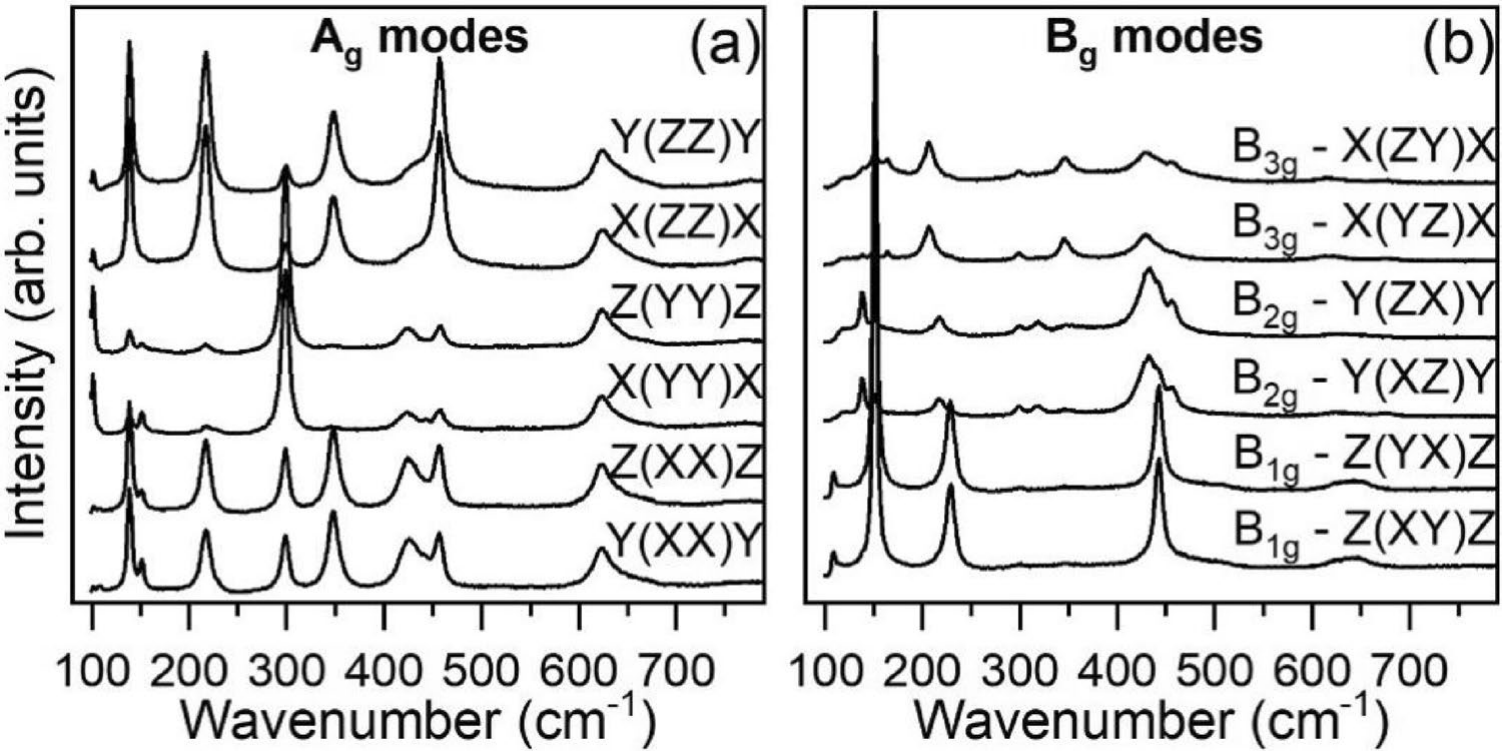
Polarized Raman spectra of NdFeO_3_ at ambient conditions. The measurement configurations are given in Porto’s notation, in (**a**) for the vibration modes of A_g_ symmetry and in (**b**) for the vibration modes of B_1,2,3g_ symmetries. X, Y and Z correspond to the orthorhombic axes in the *Pbnm* space group.

**Figure 2:**
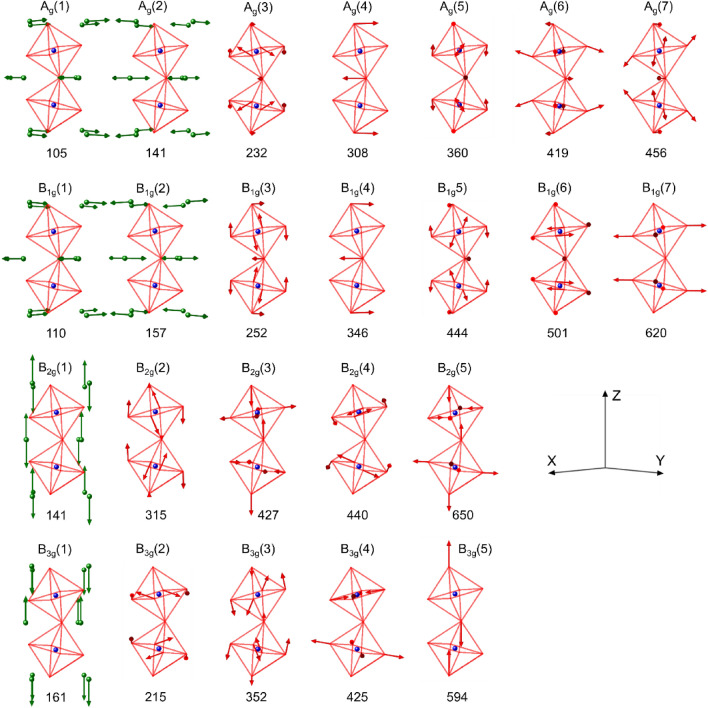
Vibrational patterns of the Raman-active modes in NdFeO_3_, described in Table 2. Label: Nd atoms (green symbols), Fe atoms (blue symbols) and O atoms (red octahedra cage).

Figure 1a shows the polarized Raman spectra for A_g_ modes, while Fig. 1b shows the polarized Raman spectra of the B_1g_, B_2g_ and B_3g_ phonons, recorded at room temperature. From the fitting procedure using Eq. 3, we have determined the wavenumber of the observed Raman-active phonons. Table 1 presents the comparison between the calculated and experimental phonon frequencies observed at 300 K and 10 K, along with the main atomic motions. The frequency of the observed phonons agrees with the predicted values obtained from our DFT calculations. At 300 K, 7 Ag phonons are observed in the 100–500 cm^−1^ spectral range, while 5 B_1g_, 5 B_2g_ and 5 B_3g_ phonons are detected in the 100–700 cm^−1^ range. At 10 K, we were successful to detect all the predicted B_1g_, B_2g_ and B_3g_ phonons. However, one of the A_g_ modes, indicated with *, was not detected. Probably this mode still exits at 10 K, but its wavenumber is below (< 100 cm^−1^) the spectral range detection limit used. Figure 2 presents a schematic view of the main atomic motion of the Raman-active modes, which is in agreement with the literature^27^. The overall results presented in this work provide a more complete description and understanding of the Raman-active phonons. The broad feature observed in the 600–700 cm^−1^ range in Fig. 1a is assigned to second order processes, as reported in other rare-earth orthoferrites^28,29^ and in agreement with the lack of predicted A_g_ modes in that frequency range (cf. Table 1). The small features observed in Fig. 1b for the B_1g_ modes, between 300 and 400 cm^−1^ are bands arising from leakage of strong A_g_ modes through the polarizer and misalignments of the samples.

**Table 1:** Experimental and theoretical band positions, the corresponding symmetry assignment and main atomic motions (identified from DFT calculations) of the observed Raman-active modes in NdFeO_3_.

Symmetry	DFT (cm^−1^)	Experimental (cm^−1^)		Main atomic motion
		10 K^5^	300 K	
A_g_	104.6 141.4 231.5 307.5 359.8 418.8 456.2	–* 142.2 217.8 305.0 357.0 422.1 460.4	101.2 138.9 216.8 298.7 348.7 425.8 457.2	Nd(y), in-phase in x–y, out-of-phase in z Nd(x), out-of-phase [001]_pc_ FeO_6_ rotation, in-phase O1 x–y plane [110]_pc_ FeO_6_ rotation, in-phase Fe-O2 stretching, in-phase O1-Fe-O2 scissor-like bending
B_1g_	110.0 157.3 251.9 346.0 443.6 500.8 619.8	109.0 153.9 233.7 320.8 448.2 504.5 620.4	109.3 151.4 228.6 – 442.5 – 628.0	Nd(x), in-phase in x–y, out-of-phase in z Nd(y), out-of-phase [110]_pc_ FeO_6_ rotation, in-phase O1 x–y plane O1-Fe-O2 scissor-like bending O2–Fe–O2 scissor-like bending, in-phase Fe–O2 stretching in-phase
B_2g_	140.9 315.4 426.6 439.9 650.4	139.6 305.8 431.5 436.8 643.0	138.3 299.2 431.4 443.1 647.5	Nd(z) out-of-phase in x–y, z O1-Fe-O2 in-phase octahedra squeezing in z O2-Fe-O2 scissor-like bending, out-of-phase FeO_6_ breathing
B_3g_	160.7 214.7 352.1 425.3 593.8	155.4 208.8 352.7 426.2 596.3	150.9 206.9 346.5 431.5 618.4	Nd(z) in-phase in x–y, out-of-phase in z [001]_pc_ FeO_6_ rotation, out-of-phase [001]_pc_ FeO_6_ rotation, out-of-phase Fe-O2 stretching, out-of-phase Fe-O1 stretching

The values at 10 K were taken from^5^.

The characterization of the Raman-active phonons of NdFeO_3_ at room temperature was already presented by Singh et al.^6^. However, this characterization has some problems. First, the theoretical model used to compute the Raman-active modes wavenumbers gives large differences between the calculated and experimental values, with the experimental values being very close to the ones presented in our work. In this regard, the first-principles calculations performed by us are more suitable to describe NdFeO_3_ phonons. Second, Singh et al.^6^ only provides the main atomic motions for the A_g_ and B_1g_ modes, whereas we provide the eigenmodes for all symmetries. Also, Singh et al. assign three Raman-active modes to breathing modes: A_g_(3), B_1g_(3) and B_1g_(7)^6^. However, as Fig. 2 demonstrates, the only breathing mode is B_2g_(5). Both A_g_(3) and B_1g_(3) are rotations and B_1g_(7) a Fe-O2 stretching mode. Finally, our results fill the gap of the Raman phonon wavenumbers of *R*FeO_3_ as a function of the rare-earth *R*^3+^ ionic radius, presented in^29^, confirming the accuracy of our analysis (see Supplemental Information).

### Infrared active phonons

**Figure 3:**
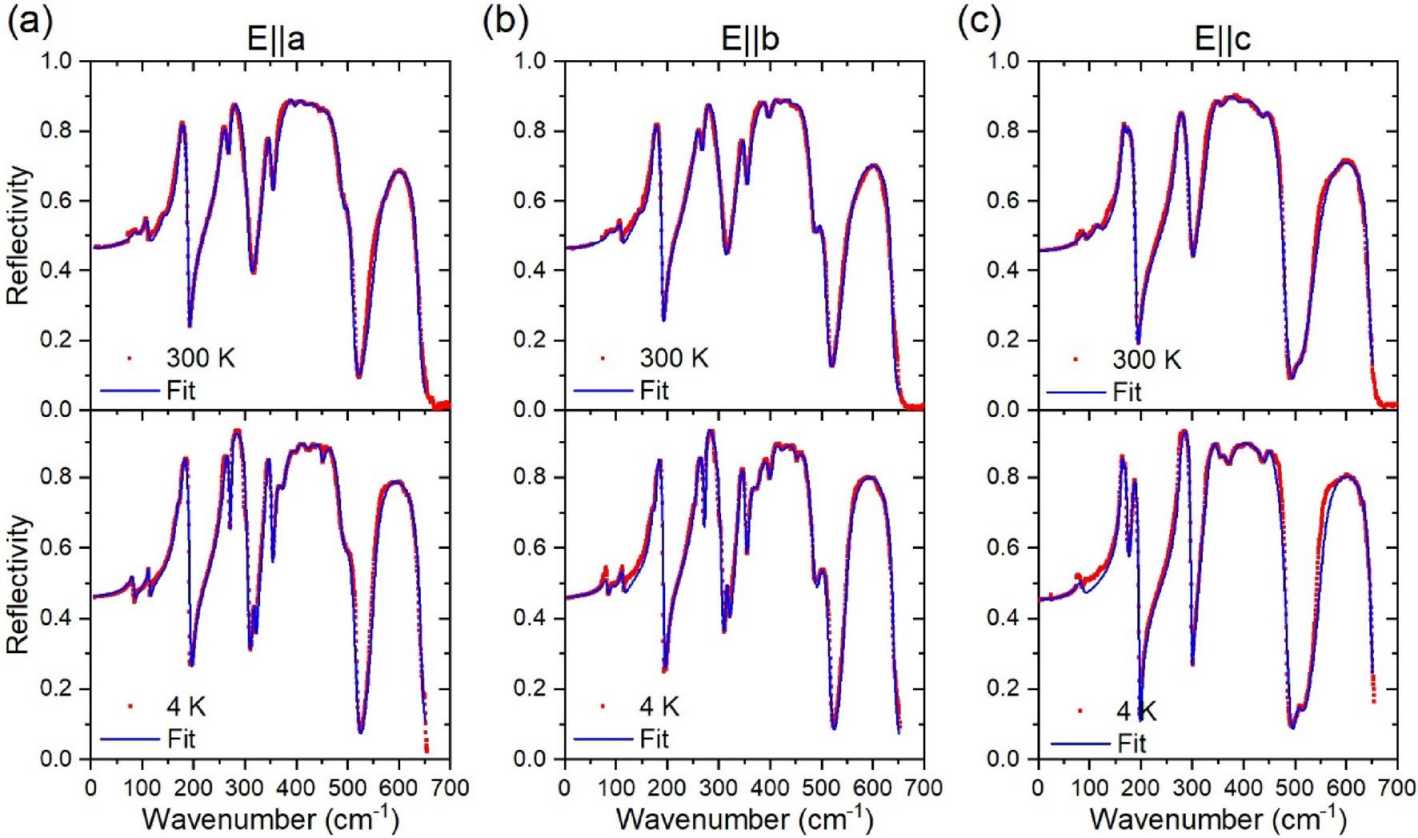
Reflectivity spectra of NdFeO_3_ recorded at 300 K and 4 K, for the polarizations: (a) E//a–B_3u_, (**b**) E//b–B_2u_ and (**c**) E//c–B_1u_. The blue solid line was determined by the best fit of Eq. 2 to the experimental reflectivity.

Figures 3(a)-(c) shows the IR reflectivity spectra recorded at 300 K and 4 K, for the IR electric vector E polarized along the a-, b- and c-axis, respectively, in the 5–700 cm^−1^ spectral range. The solid blue lines were determined by the best fit of Eqs. 1 and 2 to the experimental spectra. To perform the fits, the high frequency contributions to the dielectric function, ${\varepsilon }_{\infty }$, was taken to be 5.36 for E//a, 5.53 for E//b, and 5.34 for E//c. These values are compatible with the refractive index usually found in rare-earth orthoferrites^30^.

The room temperature reflectivity spectra are described by a total of 13 (B_3u_–E//a), 13 (B_2u_–E//b) and 11 (B_1u_–E//c) excitations, i.e., slightly more than expected from factor-group analysis in Eq. 4. Among these excitations, we found an excellent agreement between the calculated transversal optical phonon frequencies and the experimental ones at 4 K, as presented in Table 2. While we succeeded in observing all the predicted polar phonons at 4 K, the bands are broader and overlap at 300 K, preventing to resolve all modes. The remaining redundant excitations are assigned to crystal-field excitations (see section d) and leakages from other polarizations due to small misalignments of the samples (see Supplemental Information). The calculated and experimental frequencies at 4 K are in good agreement with the *Pbnm* symmetry of NdFeO_3_ at this temperature.

**Table 2:** Experimental and theoretical (only for transversal phonons) band positions, the corresponding symmetry assignment of the observed transversal and longitudinal IR-active modes in NdFeO_3_.

	Transversal (cm^−1^)			Longitudinal (cm^−1^)		Main atomic motion
	DFT	4 K	300 K	4 K	300 K	
B_1u_ (E//c)	167.3 176.4 271.8 301.7 337.6 503.8 535.9	167.5 183.1 276.5 303.6 329.5 505.4 549.3	165.0 172.6 272.0 – 330.7 506.6 540.9	174.4 197.0 298.2 304.8 350.0 510.8 648.1	170.8 191.8 297.0 – 354.8 507.8 644.5	Nd(z); Fe(y) out-of-phase in z, in-phase in x–y Nd(z) in-phase in x; Fe + O1 y–z plane O2(z) out-of-phase in z, in-phase in x–y; Fe(xyz) Fe(x) out-of-phase in z [001]_pc_ FeO_6_ rotation, out-of-phase; Fe(xyz) O2-Fe-O2 scissor-like bending, out-of-phase; Fe(y) Fe-O1 stretching; Fe(z)
B_2u_ (E//b)	110.3 189.3 242.9 282.4 302.4 322.1 405.6 503.7 520.8	112.3 179.8 259.6 275.3 315.6 340.3 401.8 492.7 548.1	109.6 174.1 255.4 272.3 – 342.7 397.0 490.9 543.3	113.7 193.7 271.7 307.2 318.6 352.4 419.2 518.0 639.7	111.3 190.3 266.8 307.8 – 353.0 413.8 512.0 636.1	Nd(x) in-phase in z, out-of-phase in x–y; Fe(x) Nd(y) against Fe O1-Fe-O2 in-phase; Fe(y) O1 against Fe in x–y plane, in-phase O2-Fe-O2 scissor-like bending; Fe(xyz) O2-Fe-O2 scissor-like bending; Fe(xz) O1 against Fe in x–y plane Fe-O2 stretching in-phase; Fe(z) Fe-O2 stretching, out-of-phase; Fe(yz)
B_3u_ (E//a)	115.0 177.2 256.4 277.3 312.2 342.5 370.3 448.3 543.8	113.8 178.3 260.8 274.1 319.2 340.9 376.5 452.4 550.6	110.2 173.5 256.6 271.7 – 341.5 358.4 439.2 549.3	114.9 194.0 260.8 307.2 321.1 352.4 400.3 489.7 642.1	112.3 190.6 267.4 308.4 – 353.0 395.5 493.3 637.3	Nd(x) in-phase in z, out-of-phase in y; Fe(y) Nd(x) against FeO_6_ octahedra [110]_pc_ FeO_6_ rotation in-phase; Fe(xyz) O1-Fe-O2 scissor-like bending; Fe(xyz) O1 x–y plane, out-of-phase; Fe(z) O1 x–y plane; Fe(yz) O1-Fe-O2 scissor-like bending; Fe(xy) O1-Fe-O2 scissor-like bending; Fe(y) Fe-O1 stretching out-of-phase; Fe(x)

**Figure 4:**
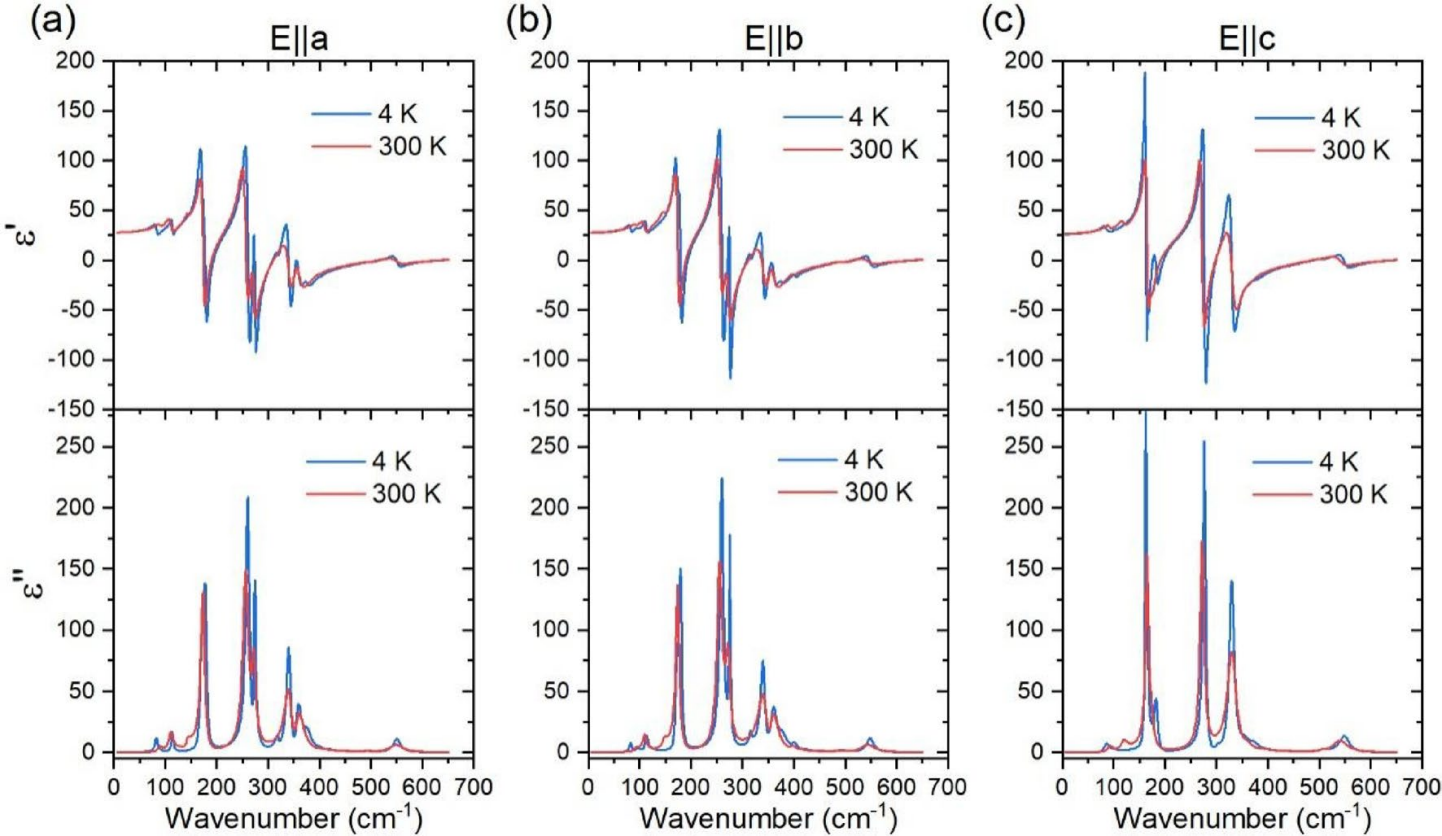
The spectra of the real $\varepsilon {\prime}$ and imaginary parts $\varepsilon {\prime}{\prime}$ of the dielectric function calculated from the reflectivity data analysis, recorded at 300 K and 4 K, for (**a**) E//a, (**b**) E//b and (**c**) E//c.

**Figure 5:**
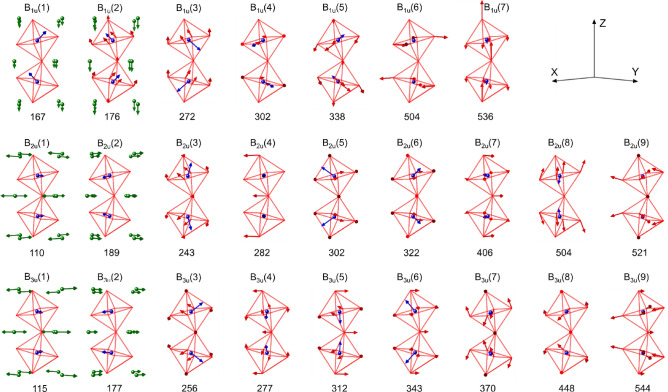
Vibrational patterns of the infrared-active modes in NdFeO_3_, described in Table 2. Label: Nd atoms (green symbols), Fe atoms (blue symbols) and O atoms (red octahedra cage).

Figure 4 shows the spectra of the real $\varepsilon {\prime}$ and imaginary parts $\varepsilon {\prime}{\prime}$ of the dielectric function, calculated from Eq. 1. Frequencies peaks in $\varepsilon {\prime}{\prime}(\omega )$ spectra roughly correspond to the frequencies of the transversal optical phonons. According to our calculations, the translational modes of Nd^3+^ and Fe^3+^ cations are observed in the 110–200 cm^−1^ spectral range, while the internal vibrations of the FeO_6_ octahedra are found to be in the 220–650 cm^−1^ range^31^. Figure 5 presents a schematic view of the main atomic motion of the IR-active modes of Table 2, which had not been reported up to now, within the best of our knowledge.

### Crystal-field excitations

Due to the crystal-field interaction, the ground state of Nd^3+^, ^9/2^I_4_, splits into 5 Kramers doublets and they can be observed in IR reflectivity spectra^32^. Following the current literature, Table 3 presents the excitation assignment to the crystal-field excitations in NdFeO_3_ at 300 K and 4 K^21^, along with the experimental values obtained from the reflectivity spectra fitting. The first doublet can be seen in all polarizations at 4 K, while the second doublet is only visible for E//a and E//b (cf. Figures 3(a)-(c)).

**Table 3:** Theoretical and experimental values of some crystal-field (CF) excitations of NdFeO_3_. Note that each CF excitation has two calculated values representing the up and down spin energies (due to Zeeman effect).

CF calculated^32^ (cm^−1^)	4 K			300 K		
	E//a	E//b	E//c	E//a	E//b	E//c
81.8–85.9	82.8	82.4	84.5	88.7	–	91.4
360.5–368.4	357.2	360.2	–	–	357.8	–

## Conclusion

We have presented a comprehensive study of the optical lattice excitations in NdFeO_3_ single crystals. The polarized Raman scattering and IR spectroscopies studies, along with the first-principles simulations, allow us the mode and symmetry assignments of the observed vibrations. In this sense, this work complements the understanding of the Raman and IR signatures of the optical phonons in NdFeO_3_ and in other rare-earth orthoferrites.

### Supplementary Information

Supplementary Information.

## Data Availability

The authors declare that the data supporting the findings of this study are available within the paper and its supplementary information file.
