# Supplementary material for: Lattice excitations in NdFeO3 through polarized optical spectroscopies
Source: Sci Rep. 2024 Jul 4;14:15378. doi: 10.1038/s41598-024-66594-w (PMC11224253; doi:10.1038/s41598-024-66594-w)
Supplement: Supplementary file 1 — Supplementary Information. [file 41598_2024_66594_MOESM1_ESM.pdf]

Supplemental Information: Lattice excitations in $NdFeO_3$ through polarized optical spectroscopies

M.M. Gomes[1], R. Vilarinho[1], H. Zhao[2], J. Íñiguez-González[3,4], M. Mihalik[5], M. Mihalik jr.[5], A. Maia[6], V. Goian[6], D. Nuzhnyy[6], S. Kamba[6] and J. Agostinho Moreira[1]

1 – IFIMUP, LaPMET, Departamento de Física e Astronomia da Faculdade de Ciências, Universidade do Porto, Rua do Campo Alegre s/n, 4169-007 Porto, Portugal

2 – Key Laboratory of Material Simulation Methods and Software of Ministry of Education, College of Physics, Jilin University, Changchun, 130012, China

3 – Materials Research and Technology Department, Luxembourg Institute of Science and Technology, 5 Avenue des Hauts-Fourneaux, L-4362 Esch/Alzette, Luxembourg

4 – Department of Physics and Materials Science, University of Luxembourg, 41 Rue du Brill, L-4422 Belvaux, Luxembourg

5 – Institute of Experimental Physics, Slovak Academy of Sciences, Watsonova 47, 040 01 Košice, Slovak Republic

6 – Institute of Physics of the Czech Academy of Sciences, Na Slovance 2, 182 00 Prague 8, Czech Republic

a. IR reflectivity spectra analysis

As mentioned in the main text, the reflectivity spectra are described by a slightly higher number of excitations than expected from factor-group analysis in Equation 4. Some of these excitations are assigned to crystal-field excitations (see section d), others to leakages from other polarizations due to small misalignments of the samples and others can be assigned to multiphonon scattering processes. Table S1 shows the parameters obtained from the IR reflectivity spectra fitting.

Table S1. Parameters obtained from the IR reflectivity spectra fitting and corresponding symmetry assignment.

**Symmetry**	**4 K**				**300 K**				**Assignment**
	ω_{TO}	γ_{TO}	ω_{LO}	γ_{LO}	ω_{TO}	γ_{TO}	ω_{LO}	γ_{LO}	
**E//a**	82.8	7.4	84.2	7.5	88.7	11.6	89.3	12.3	CF
	113.8	4.5	114.9	4.6	110.2	10.1	112.3	11.3	$B_{3u}(1)$
	170.2	8.2	172.9	12.0	142.5	8.3	142.8	9.4	Leakage from $B_{1u}(1)$
	178.3	7.1	194.0	6.1	173.5	8.8	190.6	5.6	$B_{3u}(2)$
	260.8	8.7	270.5	3.4	256.6	12.6	267.4	8.9	$B_{3u}(3)$
	274.1	4.1	307.2	8.9	271.67	8.8	308.4	18.0	$B_{3u}(4)$
	319.2	7.0	321.1	4.9	---	---	---	---	$B_{3u}(5)$
	340.9	10.4	352.4	4.6	341.5	18.3	353.0	8.7	$B_{3u}(6)$
	357.2	13.1	374.1	14.2	---	---	---	---	CF
	376.5	14.2	400.3	10.7	358.4	18.6	395.5	13.4	$B_{3u}(7)$
	400.6	10.6	425.8	11.9	395.7	14.7	413.8	19.1	Leakage from $B_{2u}(7)$
	426.0	11.6	438.5	15.2	413.8	20.5	439.1	16.3	Multiphonon scattering
	438.7	14.5	451.5	13.7	---	---	---	---	Multiphonon scattering
	452.4	13.8	489.7	22.2	439.2	16.8	493.3	18.4	$B_{3u}(8)$
	493.3	25.2	518.0	14.6	495.7	15.8	512.0	16.8	Leakage from $B_{1u}(6)$
	550.6	17.4	642.1	8.7	549.3	32.5	637.3	8.6	$B_{3u}(9)$
**E//b**	82.4	7.3	83.3	7.2	---	---	---	---	CF
	95.3	11.5	95.9	12.0	90.1	11.5	90.4	12.2	Multiphonon scattering
	112.3	6.8	113.7	8.0	109.6	9.3	111.3	9.4	$B_{2u}(1)$
	171.1	6.8	172.9	9.6	147.0	9.9	147.6	10.7	Leakage from $B_{1u}(1)$
	179.8	5.8	193.7	5.2	174.1	8.1	190.3	5.8	$B_{2u}(2)$
	259.6	8.7	271.7	4.4	255.4	12.0	266.8	12.1	$B_{2u}(3)$
	275.3	2.9	307.2	10.3	272.3	10.3	307.8	21.9	$B_{2u}(4)$
	315.6	6.4	318.6	8.3	---	---	---	---	$B_{2u}(5)$
	340.3	9.6	352.4	8.3	342.7	20.8	353.0	8.9	$B_{2u}(6)$
	360.2	14.1	372.3	19.8	357.8	19.6	395.9	12.6	CF
	376.5	23.3	400.0	12.8	---	---	---	---	Leakage from $B_{3u}(7)$
	401.8	13.0	419.2	20.5	397.0	14.1	413.8	18.8	$B_{2u}(7)$
	419.8	22.3	451.8	15.5	413.8	20.4	439.1	16.3	Multiphonon scattering
	452.4	15.7	486.1	19.9	439.2	16.8	484.9	21.1	Leakage from $B_{3u}(8)$
	492.7	24.3	518.0	14.3	490.9	23.2	512.0	17.1	$B_{2u}(8)$
	548.1	17.2	639.7	7.5	543.3	32.2	636.1	7.3	$B_{2u}(9)$
**E//c**	84.5	12.6	85.8	14.1	91.4	11.2	92.2	11.9	CF
	162.3	3.3	166.6	4.7	119.3	13.9	120.6	15.0	Leakage from $B_{3u}(2)$
	167.5	4.3	174.4	8.3	165.0	7.0	170.8	8.8	$B_{1u}(1)$
	183.1	9.0	197.0	3.2	172.6	10.4	191.8	7.0	$B_{1u}(2)$
	276.5	6.0	298.2	3.8	272.0	9.3	297.0	14.6	$B_{1u}(3)$
	303.6	10.3	304.8	8.9	---	---	---	---	$B_{1u}(4)$
	329.5	11.8	350.0	14.4	330.7	20.9	354.8	19.7	$B_{1u}(5)$
	350.6	17.0	371.0	20.8	355.4	22.9	384.3	26.1	Leakage from $B_{3u}(6)$
	372.9	21.3	445.7	15.2	384.4	28.2	438.7	16.1	Leakage from $B_{3u}(7)$
	445.8	13.9	487.9	15.8	439.1	15.3	484.3	19.5	Leakage from $B_{3u}(8)$
	505.4	18.9	510.8	22.6	506.6	11.6	507.8	11.1	$B_{1u}(6)$
	549.3	23.1	648.1	7.4	540.9	35.0	644.5	9.4	$B_{1u}(7)$

b. *R*FeO₃ Raman modes vs. ionic radius

The Raman analysis in $NdFeO_3$ complements the study already done for the other rare-earth orthoferrites [29]. Figure S1 presents the wavenumber of selected Raman-active phonons as a function of the ionic radius, combining the data of Ref.[29] and with the one of this work. As it can be ascertained from this figure, our data fits very well the ionic radius trend of the Raman-active phonon wavenumbers in the $RFeO_3$ family, with R = Dy – La.

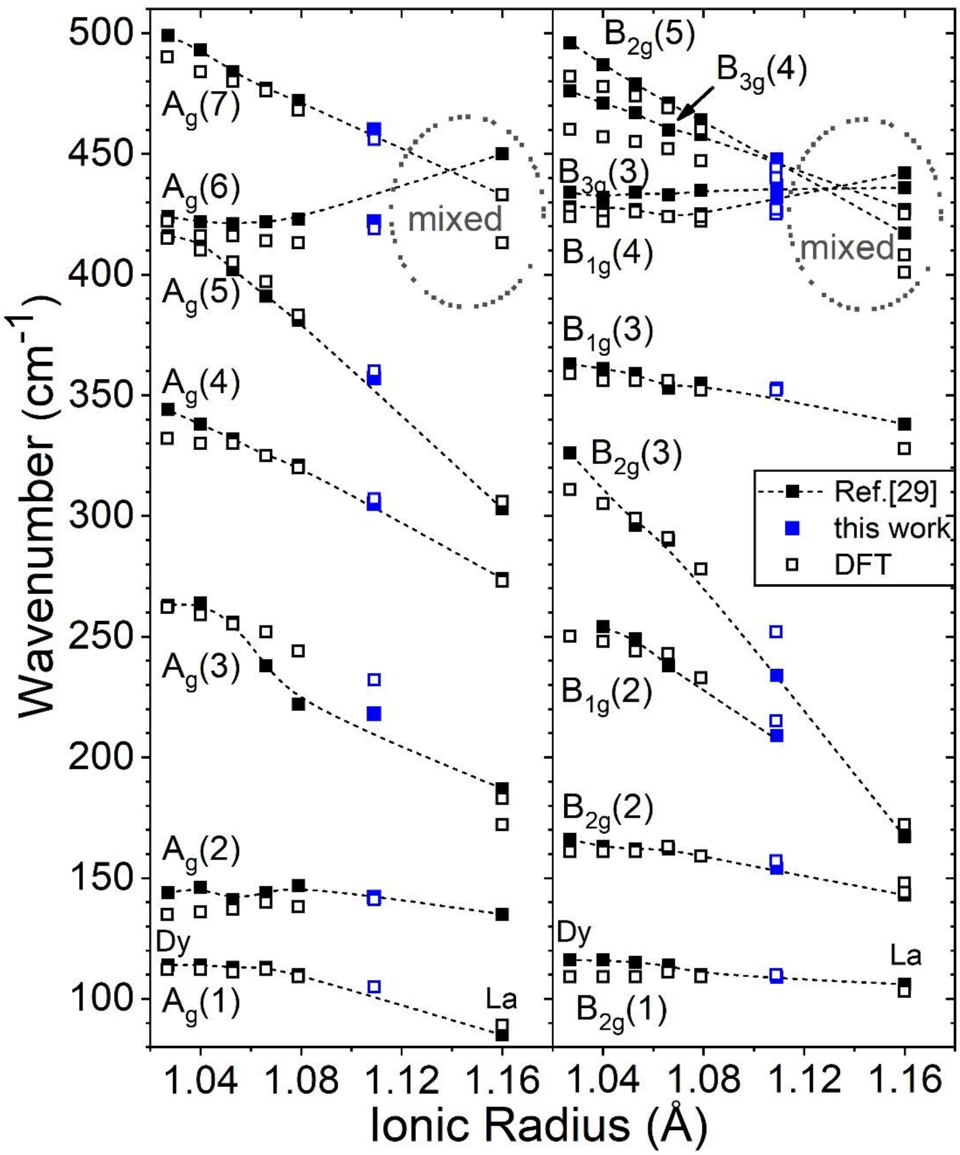

Figure S1. Raman phonon wavenumbers of $RFeO_3$ as a function of the rare earth R^{3+} ionic radius. The data for all rare-earth was take from Ref.[29], with the exception of Nd (this work, blue data). All lines are guides to the eye only. The setting used in this figure is *Pnma* space group, for an easier comparison with Ref.[29] .

c. Phonon file obtained from DFT calculations using VASP software

The following 60 tables represent the zone-center phonons (one table per phonon), listed sequentially from higher to lower energy.

The first row gives the phonon assignment and frequency in various units. The columns labelled X, Y and Z give the Cartesian coordinates of the atoms in the ground state structure, in angstroms. The columns labelled dx, dy and dz are the phonon eigenmode, i.e., the corresponding spatial displacements atom by atom. For each phonon, the 20 atoms in the unit cell are listed in the following order: 4 Nd atoms, 4 Fe atoms, and 12 O atoms. Please note that these tables are given for the Pnma setting. In the main text, the Pbnm setting was used, so a permutation of the coordinates and respective displacements must be taken into account. Assignment motions names are in the Pbnm setting to be coherent with main text.

$B_2g(5)$, FeO_6 breathing

1 f = 19.497811 THz 122.508362 2PiTHz 650.376963 cm-1 80.636495 meV

X	Y	Z	dx	dy	dz
0.303633	1.932008	0.073534	-0.000000	-0.002648	-0.000000
5.287376	5.796024	5.341894	0.000000	0.002648	-0.000000
2.491871	5.796024	2.781249	-0.000000	-0.002648	0.000000
3.099138	1.932008	2.634180	-0.000000	0.002648	0.000000
0.000000	0.000000	2.707714	-0.000000	-0.000000	-0.000000
2.795505	0.000000	0.000000	-0.000000	0.000000	-0.000000
0.000000	3.864016	2.707714	0.000000	0.000000	0.000000
2.795505	3.864016	0.000000	0.000000	0.000000	0.000000
2.659869	1.932008	4.933751	0.000000	0.301742	0.000000
2.931141	5.796024	0.481677	0.000000	-0.301742	-0.000000
0.135636	5.796024	2.226037	-0.000000	0.301742	-0.000000
5.455373	1.932008	3.189392	-0.000000	-0.301742	0.000000
4.440201	0.366692	1.112822	0.210200	0.014126	0.187320
1.150808	7.361340	4.302607	-0.210200	-0.014126	-0.187320
3.946313	7.361340	3.820536	0.210200	0.014126	-0.187320
1.644697	0.366692	1.594893	-0.210200	-0.014126	0.187320
1.150808	4.230709	4.302607	0.210200	-0.014126	0.187320

4.440201	3.497324	1.112822	-0.210200	0.014126	-0.187320
1.644697	3.497324	1.594893	0.210200	-0.014126	-0.187320
3.946313	4.230709	3.820536	-0.210200	0.014126	0.187320

--

B_{1g}(7), Fe-O2 stretching in-phase

2 f = 18.580834 THz 116.746823 2PiTHz 619.789887 cm-1 76.844180 meV

X	Y	Z	dx	dy	dz
0.303633	1.932008	0.073534	-0.011887	-0.000000	-0.006654
5.287376	5.796024	5.341894	0.011887	0.000000	0.006654
2.491871	5.796024	2.781249	-0.011887	0.000000	0.006654
3.099138	1.932008	2.634180	0.011887	-0.000000	-0.006654
0.000000	0.000000	2.707714	0.000000	-0.000000	0.000000
2.795505	0.000000	0.000000	0.000000	-0.000000	0.000000
0.000000	3.864016	2.707714	-0.000000	0.000000	0.000000
2.795505	3.864016	0.000000	0.000000	0.000000	-0.000000
2.659869	1.932008	4.933751	0.017952	0.000000	0.096306
2.931141	5.796024	0.481677	-0.017952	-0.000000	-0.096306
0.135636	5.796024	2.226037	0.017952	-0.000000	-0.096306
5.455373	1.932008	3.189392	-0.017952	0.000000	0.096306
4.440201	0.366692	1.112822	-0.254175	-0.002058	-0.235584
1.150808	7.361340	4.302607	0.254175	0.002058	0.235584
3.946313	7.361340	3.820536	-0.254175	-0.002058	0.235584
1.644697	0.366692	1.594893	0.254175	0.002058	-0.235584
1.150808	4.230709	4.302607	0.254175	-0.002058	0.235584
4.440201	3.497324	1.112822	-0.254175	0.002058	-0.235584
1.644697	3.497324	1.594893	0.254175	-0.002058	-0.235584
3.946313	4.230709	3.820536	-0.254175	0.002058	0.235584

--

B_{3g}(5), Fe-O1 stretching

3 f = 17.802914 THz 111.859006 2PiTHz 593.841263 cm-1 73.626959 meV

X	Y	Z	dx	dy	dz
0.303633	1.932008	0.073534	0.000000	-0.071247	-0.000000
5.287376	5.796024	5.341894	-0.000000	0.071247	0.000000
2.491871	5.796024	2.781249	-0.000000	0.071247	-0.000000
3.099138	1.932008	2.634180	-0.000000	-0.071247	0.000000
0.000000	0.000000	2.707714	-0.000000	0.000000	0.000000
2.795505	0.000000	0.000000	-0.000000	0.000000	0.000000
0.000000	3.864016	2.707714	-0.000000	-0.000000	0.000000
2.795505	3.864016	0.000000	-0.000000	-0.000000	0.000000
2.659869	1.932008	4.933751	-0.000000	0.468690	0.000000
2.931141	5.796024	0.481677	0.000000	-0.468690	0.000000
0.135636	5.796024	2.226037	-0.000000	-0.468690	0.000000
5.455373	1.932008	3.189392	-0.000000	0.468690	0.000000
4.440201	0.366692	1.112822	0.036330	0.101412	-0.031972
1.150808	7.361340	4.302607	-0.036330	-0.101412	0.031972
3.946313	7.361340	3.820536	-0.036330	-0.101412	-0.031972
1.644697	0.366692	1.594893	0.036330	0.101412	0.031972
1.150808	4.230709	4.302607	0.036330	-0.101412	-0.031972
4.440201	3.497324	1.112822	-0.036330	0.101412	0.031972
1.644697	3.497324	1.594893	-0.036330	0.101412	-0.031972
3.946313	4.230709	3.820536	0.036330	-0.101412	0.031972

--

B_{3u}(9), Fe-O1 stretching out-of-phase, Fe(x)

4 f = 16.302061 THz 102.428873 2PiTHz 543.778221 cm-1 67.419931 meV

X	Y	Z	dx	dy	dz
0.303633	1.932008	0.073534	-0.022211	-0.000000	0.012519
5.287376	5.796024	5.341894	-0.022211	-0.000000	0.012519
2.491871	5.796024	2.781249	0.022211	0.000000	0.012519

3.099138	1.932008	2.634180	0.022211	-0.000000	0.012519
0.000000	0.000000	2.707714	-0.021672	0.010557	-0.072974
2.795505	0.000000	0.000000	0.021672	-0.010557	-0.072974
0.000000	3.864016	2.707714	-0.021672	-0.010557	-0.072974
2.795505	3.864016	0.000000	0.021672	0.010557	-0.072974
2.659869	1.932008	4.933751	-0.057633	-0.000000	-0.157225
2.931141	5.796024	0.481677	-0.057633	-0.000000	-0.157225
0.135636	5.796024	2.226037	0.057633	-0.000000	-0.157225
5.455373	1.932008	3.189392	0.057633	0.000000	-0.157225
4.440201	0.366692	1.112822	0.296080	0.060618	0.127918
1.150808	7.361340	4.302607	0.296080	0.060618	0.127918
3.946313	7.361340	3.820536	-0.296080	-0.060618	0.127918
1.644697	0.366692	1.594893	-0.296080	-0.060618	0.127918
1.150808	4.230709	4.302607	0.296080	-0.060618	0.127918
4.440201	3.497324	1.112822	0.296080	-0.060618	0.127918
1.644697	3.497324	1.594893	-0.296080	0.060618	0.127918
3.946313	4.230709	3.820536	-0.296080	0.060618	0.127918

--

B_{1u}(7), Fe-O1 stretching; Fe(z)

5 f = 16.065939 THz 100.945274 2PiTHz 535.902037 cm-1 66.443408 meV

X	Y	Z	dx	dy	dz
0.303633	1.932008	0.073534	-0.000000	0.003215	0.000000
5.287376	5.796024	5.341894	-0.000000	0.003215	0.000000
2.491871	5.796024	2.781249	-0.000000	0.003215	-0.000000
3.099138	1.932008	2.634180	-0.000000	0.003215	-0.000000
0.000000	0.000000	2.707714	0.013504	-0.099035	-0.002977
2.795505	0.000000	0.000000	0.013504	-0.099035	0.002977
0.000000	3.864016	2.707714	-0.013504	-0.099035	0.002977
2.795505	3.864016	0.000000	-0.013504	-0.099035	-0.002977

2.659869	1.932008	4.933751	0.000000	0.448457	-0.000000
2.931141	5.796024	0.481677	0.000000	0.448457	-0.000000
0.135636	5.796024	2.226037	0.000000	0.448457	0.000000
5.455373	1.932008	3.189392	0.000000	0.448457	0.000000
4.440201	0.366692	1.112822	-0.025460	-0.136490	0.012677
1.150808	7.361340	4.302607	-0.025460	-0.136490	0.012677
3.946313	7.361340	3.820536	-0.025460	-0.136490	-0.012677
1.644697	0.366692	1.594893	-0.025460	-0.136490	-0.012677
1.150808	4.230709	4.302607	0.025460	-0.136490	-0.012677
4.440201	3.497324	1.112822	0.025460	-0.136490	-0.012677
1.644697	3.497324	1.594893	0.025460	-0.136490	0.012677
3.946313	4.230709	3.820536	0.025460	-0.136490	0.012677

--

A_u(8) silent mode

6 f = 15.636341 THz 98.246029 2PiTHz 521.572183 cm-1 64.666732 meV

X	Y	Z	dx	dy	dz
0.303633	1.932008	0.073534	-0.000000	-0.014345	0.000000
5.287376	5.796024	5.341894	-0.000000	-0.014345	0.000000
2.491871	5.796024	2.781249	-0.000000	0.014345	-0.000000
3.099138	1.932008	2.634180	-0.000000	0.014345	-0.000000
0.000000	0.000000	2.707714	-0.011732	-0.028448	-0.084832
2.795505	0.000000	0.000000	0.011732	0.028448	-0.084832
0.000000	3.864016	2.707714	0.011732	-0.028448	0.084832
2.795505	3.864016	0.000000	-0.011732	0.028448	0.084832
2.659869	1.932008	4.933751	0.000000	-0.200254	-0.000000
2.931141	5.796024	0.481677	0.000000	-0.200254	-0.000000
0.135636	5.796024	2.226037	0.000000	0.200254	0.000000
5.455373	1.932008	3.189392	0.000000	0.200254	0.000000
4.440201	0.366692	1.112822	0.296833	0.037936	0.105952

1.150808	7.361340	4.302607	0.296833	0.037936	0.105952
3.946313	7.361340	3.820536	-0.296833	-0.037936	0.105952
1.644697	0.366692	1.594893	-0.296833	-0.037936	0.105952
1.150808	4.230709	4.302607	-0.296833	0.037936	-0.105952
4.440201	3.497324	1.112822	-0.296833	0.037936	-0.105952
1.644697	3.497324	1.594893	0.296833	-0.037936	-0.105952
3.946313	4.230709	3.820536	0.296833	-0.037936	-0.105952

--

B_{2u}(9), Fe-O2 stretching, out-of-phase; Fe(yz)

7 f = 15.613641 THz 98.103398 2PiTHz 520.814980 cm-1 64.572851 meV

X	Y	Z	dx	dy	dz
0.303633	1.932008	0.073534	-0.013228	0.000000	0.012379
5.287376	5.796024	5.341894	-0.013228	0.000000	0.012379
2.491871	5.796024	2.781249	-0.013228	-0.000000	-0.012379
3.099138	1.932008	2.634180	-0.013228	-0.000000	-0.012379
0.000000	0.000000	2.707714	0.078546	-0.072053	0.010113
2.795505	0.000000	0.000000	0.078546	-0.072053	-0.010113
0.000000	3.864016	2.707714	0.078546	0.072053	0.010113
2.795505	3.864016	0.000000	0.078546	0.072053	-0.010113
2.659869	1.932008	4.933751	0.139611	0.000000	-0.111290
2.931141	5.796024	0.481677	0.139611	0.000000	-0.111290
0.135636	5.796024	2.226037	0.139611	-0.000000	0.111290
5.455373	1.932008	3.189392	0.139611	-0.000000	0.111290
4.440201	0.366692	1.112822	-0.123206	0.088241	-0.283195
1.150808	7.361340	4.302607	-0.123206	0.088241	-0.283195
3.946313	7.361340	3.820536	-0.123206	0.088241	0.283195
1.644697	0.366692	1.594893	-0.123206	0.088241	0.283195
1.150808	4.230709	4.302607	-0.123206	-0.088241	-0.283195
4.440201	3.497324	1.112822	-0.123206	-0.088241	-0.283195

1.644697	3.497324	1.594893	-0.123206	-0.088241	0.283195
3.946313	4.230709	3.820536	-0.123206	-0.088241	0.283195

--

A_u(7) silent mode

8 f = 15.200601 THz 95.508191 2PiTHz 507.037445 cm-1 62.864654 meV

X	Y	Z	dx	dy	dz
0.303633	1.932008	0.073534	0.000000	0.003647	0.000000
5.287376	5.796024	5.341894	-0.000000	0.003647	-0.000000
2.491871	5.796024	2.781249	-0.000000	-0.003647	0.000000
3.099138	1.932008	2.634180	0.000000	-0.003647	0.000000
0.000000	0.000000	2.707714	0.014309	-0.124106	0.020614
2.795505	0.000000	0.000000	-0.014309	0.124106	0.020614
0.000000	3.864016	2.707714	-0.014309	-0.124106	-0.020614
2.795505	3.864016	0.000000	0.014309	0.124106	-0.020614
2.659869	1.932008	4.933751	0.000000	-0.437199	0.000000
2.931141	5.796024	0.481677	0.000000	-0.437199	-0.000000
0.135636	5.796024	2.226037	0.000000	0.437199	-0.000000
5.455373	1.932008	3.189392	0.000000	0.437199	0.000000
4.440201	0.366692	1.112822	-0.118146	-0.011019	-0.085592
1.150808	7.361340	4.302607	-0.118146	-0.011019	-0.085592
3.946313	7.361340	3.820536	0.118146	0.011019	-0.085592
1.644697	0.366692	1.594893	0.118146	0.011019	-0.085592
1.150808	4.230709	4.302607	0.118146	-0.011019	0.085592
4.440201	3.497324	1.112822	0.118146	-0.011019	0.085592
1.644697	3.497324	1.594893	-0.118146	0.011019	0.085592
3.946313	4.230709	3.820536	-0.118146	0.011019	0.085592

--

B_{1u}(6), O2-Fe-O2 scissor-like bending, out-of-phase, Fe(y)

9 f = 15.104461 THz 94.904126 2PiTHz 503.830563 cm-1 62.467051 meV

X	Y	Z	dx	dy	dz
0.303633	1.932008	0.073534	-0.000000	0.009866	0.000000
5.287376	5.796024	5.341894	-0.000000	0.009866	0.000000
2.491871	5.796024	2.781249	-0.000000	0.009866	-0.000000
3.099138	1.932008	2.634180	-0.000000	0.009866	-0.000000
0.000000	0.000000	2.707714	-0.093023	0.015530	-0.026883
2.795505	0.000000	0.000000	-0.093023	0.015530	0.026883
0.000000	3.864016	2.707714	0.093023	0.015530	0.026883
2.795505	3.864016	0.000000	0.093023	0.015530	-0.026883
2.659869	1.932008	4.933751	0.000000	-0.010525	-0.000000
2.931141	5.796024	0.481677	-0.000000	-0.010525	0.000000
0.135636	5.796024	2.226037	0.000000	-0.010525	0.000000
5.455373	1.932008	3.189392	-0.000000	-0.010525	-0.000000
4.440201	0.366692	1.112822	0.141022	-0.024108	0.315625
1.150808	7.361340	4.302607	0.141022	-0.024108	0.315625
3.946313	7.361340	3.820536	0.141022	-0.024108	-0.315625
1.644697	0.366692	1.594893	0.141022	-0.024108	-0.315625
1.150808	4.230709	4.302607	-0.141022	-0.024108	-0.315625
4.440201	3.497324	1.112822	-0.141022	-0.024108	-0.315625
1.644697	3.497324	1.594893	-0.141022	-0.024108	0.315625
3.946313	4.230709	3.820536	-0.141022	-0.024108	0.315625

--

B_{2u}(8), Fe-O(2) stretching in-phase, Fe(z)

10 f = 15.101179 THz 94.883508 2PiTHz 503.721107 cm-1 62.453480 meV

X	Y	Z	dx	dy	dz
0.303633	1.932008	0.073534	-0.026641	-0.000000	0.026233
5.287376	5.796024	5.341894	-0.026641	-0.000000	0.026233
2.491871	5.796024	2.781249	-0.026641	-0.000000	-0.026233
3.099138	1.932008	2.634180	-0.026641	-0.000000	-0.026233

0.000000	0.000000	2.707714	-0.023375	-0.203900	0.019982
2.795505	0.000000	0.000000	-0.023375	-0.203900	-0.019982
0.000000	3.864016	2.707714	-0.023375	0.203900	0.019982
2.795505	3.864016	0.000000	-0.023375	0.203900	-0.019982
2.659869	1.932008	4.933751	-0.078999	-0.000000	-0.014963
2.931141	5.796024	0.481677	-0.078999	-0.000000	-0.014963
0.135636	5.796024	2.226037	-0.078999	0.000000	0.014963
5.455373	1.932008	3.189392	-0.078999	0.000000	0.014963
4.440201	0.366692	1.112822	0.101156	0.294128	0.055356
1.150808	7.361340	4.302607	0.101156	0.294128	0.055356
3.946313	7.361340	3.820536	0.101156	0.294128	-0.055356
1.644697	0.366692	1.594893	0.101156	0.294128	-0.055356
1.150808	4.230709	4.302607	0.101156	-0.294128	0.055356
4.440201	3.497324	1.112822	0.101156	-0.294128	0.055356
1.644697	3.497324	1.594893	0.101156	-0.294128	-0.055356
3.946313	4.230709	3.820536	0.101156	-0.294128	-0.055356

--

B_{1g}(6); O2-Fe-O2 scissor-like bending, in-phase

11 f = 15.014478 THz 94.338749 2PiTHz 500.829067 cm-1 62.094913 meV

X	Y	Z	dx	dy	dz
0.303633	1.932008	0.073534	0.005430	-0.000000	-0.012385
5.287376	5.796024	5.341894	-0.005430	-0.000000	0.012385
2.491871	5.796024	2.781249	0.005430	-0.000000	0.012385
3.099138	1.932008	2.634180	-0.005430	-0.000000	-0.012385
0.000000	0.000000	2.707714	0.000000	-0.000000	0.000000
2.795505	0.000000	0.000000	0.000000	0.000000	-0.000000
0.000000	3.864016	2.707714	-0.000000	-0.000000	-0.000000
2.795505	3.864016	0.000000	-0.000000	0.000000	0.000000
2.659869	1.932008	4.933751	0.085179	-0.000000	-0.172521

2.931141	5.796024	0.481677	-0.085179	-0.000000	0.172521
0.135636	5.796024	2.226037	0.085179	0.000000	0.172521
5.455373	1.932008	3.189392	-0.085179	0.000000	-0.172521
4.440201	0.366692	1.112822	-0.236435	-0.024919	0.223331
1.150808	7.361340	4.302607	0.236435	0.024919	-0.223331
3.946313	7.361340	3.820536	-0.236435	-0.024919	-0.223331
1.644697	0.366692	1.594893	0.236435	0.024919	0.223331
1.150808	4.230709	4.302607	0.236435	-0.024919	-0.223331
4.440201	3.497324	1.112822	-0.236435	0.024919	0.223331
1.644697	3.497324	1.594893	0.236435	-0.024919	0.223331
3.946313	4.230709	3.820536	-0.236435	0.024919	-0.223331

--

A_g(7); O1-Fe-O2 scissor-like bending

12 f = 13.676042 THz 85.929108 2PiTHz 456.183656 cm-1 56.559585 meV

X	Y	Z	dx	dy	dz
0.303633	1.932008	0.073534	-0.002522	-0.000000	0.020087
5.287376	5.796024	5.341894	0.002522	0.000000	-0.020087
2.491871	5.796024	2.781249	0.002522	0.000000	0.020087
3.099138	1.932008	2.634180	-0.002522	0.000000	-0.020087
0.000000	0.000000	2.707714	0.000000	0.000000	0.000000
2.795505	0.000000	0.000000	0.000000	0.000000	-0.000000
0.000000	3.864016	2.707714	-0.000000	-0.000000	-0.000000
2.795505	3.864016	0.000000	0.000000	-0.000000	-0.000000
2.659869	1.932008	4.933751	-0.101315	-0.000000	0.327775
2.931141	5.796024	0.481677	0.101315	-0.000000	-0.327775
0.135636	5.796024	2.226037	0.101315	0.000000	0.327775
5.455373	1.932008	3.189392	-0.101315	0.000000	-0.327775
4.440201	0.366692	1.112822	0.141150	0.195804	0.087647
1.150808	7.361340	4.302607	-0.141150	-0.195804	-0.087647

3.946313	7.361340	3.820536	-0.141150	-0.195804	0.087647
1.644697	0.366692	1.594893	0.141150	0.195804	-0.087647
1.150808	4.230709	4.302607	-0.141150	0.195804	-0.087647
4.440201	3.497324	1.112822	0.141150	-0.195804	0.087647
1.644697	3.497324	1.594893	0.141150	-0.195804	-0.087647
3.946313	4.230709	3.820536	-0.141150	0.195804	0.087647

--

B_{3u}(8); O1-Fe-O2 scissor-like bending; Fe(y)

13 f = 13.439061 THz 84.440109 2PiTHz 448.278802 cm-1 55.579508 meV

X	Y	Z	dx	dy	dz
0.303633	1.932008	0.073534	0.055055	-0.000000	0.010511
5.287376	5.796024	5.341894	0.055055	-0.000000	0.010511
2.491871	5.796024	2.781249	-0.055055	0.000000	0.010511
3.099138	1.932008	2.634180	-0.055055	0.000000	0.010511
0.000000	0.000000	2.707714	0.083059	-0.003093	-0.001667
2.795505	0.000000	0.000000	-0.083059	0.003093	-0.001667
0.000000	3.864016	2.707714	0.083059	0.003093	-0.001667
2.795505	3.864016	0.000000	-0.083059	-0.003093	-0.001667
2.659869	1.932008	4.933751	0.294162	0.000000	-0.277872
2.931141	5.796024	0.481677	0.294162	-0.000000	-0.277872
0.135636	5.796024	2.226037	-0.294162	0.000000	-0.277872
5.455373	1.932008	3.189392	-0.294162	-0.000000	-0.277872
4.440201	0.366692	1.112822	-0.066802	-0.134427	0.124775
1.150808	7.361340	4.302607	-0.066802	-0.134427	0.124775
3.946313	7.361340	3.820536	0.066802	0.134427	0.124775
1.644697	0.366692	1.594893	0.066802	0.134427	0.124775
1.150808	4.230709	4.302607	-0.066802	0.134427	0.124775
4.440201	3.497324	1.112822	-0.066802	0.134427	0.124775
1.644697	3.497324	1.594893	0.066802	-0.134427	0.124775

3.946313 4.230709 3.820536   0.066802   -0.134427   0.124775

--

B_{1g}(5); O1-Fe-O2 scissor-like bending

14 f =   13.297476 THz   83.550506 2PiTHz 443.556043 cm-1   54.993960 meV

X	Y	Z	dx	dy	dz
0.303633	1.932008	0.073534	-0.008841	-0.000000	0.046697
5.287376	5.796024	5.341894	0.008841	0.000000	-0.046697
2.491871	5.796024	2.781249	-0.008841	-0.000000	-0.046697
3.099138	1.932008	2.634180	0.008841	0.000000	0.046697
0.000000	0.000000	2.707714	-0.000000	-0.000000	0.000000
2.795505	0.000000	0.000000	0.000000	-0.000000	-0.000000
0.000000	3.864016	2.707714	-0.000000	0.000000	0.000000
2.795505	3.864016	0.000000	0.000000	0.000000	-0.000000
2.659869	1.932008	4.933751	0.320768	0.000000	-0.202853
2.931141	5.796024	0.481677	-0.320768	-0.000000	0.202853
0.135636	5.796024	2.226037	0.320768	0.000000	0.202853
5.455373	1.932008	3.189392	-0.320768	-0.000000	-0.202853
4.440201	0.366692	1.112822	0.038797	0.212036	-0.073388
1.150808	7.361340	4.302607	-0.038797	-0.212036	0.073388
3.946313	7.361340	3.820536	0.038797	0.212036	0.073388
1.644697	0.366692	1.594893	-0.038797	-0.212036	-0.073388
1.150808	4.230709	4.302607	-0.038797	0.212036	0.073388
4.440201	3.497324	1.112822	0.038797	-0.212036	-0.073388
1.644697	3.497324	1.594893	-0.038797	0.212036	-0.073388
3.946313	4.230709	3.820536	0.038797	-0.212036	0.073388

--

B_{2g}(4); O2-Fe-O2 scissor-like bending, out-of-phase

15 f =   13.186809 THz   82.855163 2PiTHz 439.864579 cm-1   54.536277 meV

X Y Z dx dy dz

0.303633	1.932008	0.073534	-0.000000	0.014027	0.000000
5.287376	5.796024	5.341894	-0.000000	-0.014027	-0.000000
2.491871	5.796024	2.781249	0.000000	0.014027	-0.000000
3.099138	1.932008	2.634180	0.000000	-0.014027	0.000000
0.000000	0.000000	2.707714	-0.000000	-0.000000	0.000000
2.795505	0.000000	0.000000	0.000000	-0.000000	0.000000
0.000000	3.864016	2.707714	-0.000000	0.000000	0.000000
2.795505	3.864016	0.000000	0.000000	0.000000	-0.000000
2.659869	1.932008	4.933751	0.000000	-0.103437	-0.000000
2.931141	5.796024	0.481677	-0.000000	0.103437	0.000000
0.135636	5.796024	2.226037	0.000000	-0.103437	0.000000
5.455373	1.932008	3.189392	-0.000000	0.103437	-0.000000
4.440201	0.366692	1.112822	-0.160282	-0.137745	0.273656
1.150808	7.361340	4.302607	0.160282	0.137745	-0.273656
3.946313	7.361340	3.820536	-0.160282	-0.137745	-0.273656
1.644697	0.366692	1.594893	0.160282	0.137745	0.273656
1.150808	4.230709	4.302607	-0.160282	0.137745	0.273656
4.440201	3.497324	1.112822	0.160282	-0.137745	-0.273656
1.644697	3.497324	1.594893	-0.160282	0.137745	-0.273656
3.946313	4.230709	3.820536	0.160282	-0.137745	0.273656

--

B_{2g}(3); octahedra squeezing in z

16 f = 12.788091 THz 80.349944 2PiTHz 426.564781 cm-1 52.887311 meV

X	Y	Z	dx	dy	dz
0.303633	1.932008	0.073534	-0.000000	0.005493	-0.000000
5.287376	5.796024	5.341894	-0.000000	-0.005493	0.000000
2.491871	5.796024	2.781249	0.000000	0.005493	-0.000000
3.099138	1.932008	2.634180	0.000000	-0.005493	-0.000000
0.000000	0.000000	2.707714	-0.000000	-0.000000	-0.000000

2.795505 0.000000 0.000000 0.000000 -0.000000 0.000000

0.000000 3.864016 2.707714 -0.000000 0.000000 -0.000000

2.795505 3.864016 0.000000 0.000000 0.000000 0.000000

2.659869 1.932008 4.933751 -0.000000 0.383112 -0.000000

2.931141 5.796024 0.481677 0.000000 -0.383112 0.000000

0.135636 5.796024 2.226037 0.000000 0.383112 0.000000

5.455373 1.932008 3.189392 0.000000 -0.383112 0.000000

4.440201 0.366692 1.112822 -0.217637 -0.015723 -0.063122

1.150808 7.361340 4.302607 0.217637 0.015723 0.063122

3.946313 7.361340 3.820536 -0.217637 -0.015723 0.063122

1.644697 0.366692 1.594893 0.217637 0.015723 -0.063122

1.150808 4.230709 4.302607 -0.217637 0.015723 -0.063122

4.440201 3.497324 1.112822 0.217637 -0.015723 0.063122

1.644697 3.497324 1.594893 -0.217637 0.015723 0.063122

3.946313 4.230709 3.820536 0.217637 -0.015723 -0.063122

--

B_{3g}(4); Fe-O2 stretching, out-of-phase

17 f = 12.751324 THz 80.118934 2PiTHz 425.338386 cm-1 52.735258 meV

X Y Z dx dy dz

0.303633 1.932008 0.073534 -0.000000 0.021247 -0.000000

5.287376 5.796024 5.341894 -0.000000 -0.021247 -0.000000

2.491871 5.796024 2.781249 0.000000 -0.021247 0.000000

3.099138 1.932008 2.634180 0.000000 0.021247 0.000000

0.000000 0.000000 2.707714 0.000000 -0.000000 0.000000

2.795505 0.000000 0.000000 -0.000000 -0.000000 -0.000000

0.000000 3.864016 2.707714 -0.000000 0.000000 0.000000

2.795505 3.864016 0.000000 0.000000 0.000000 -0.000000

2.659869 1.932008 4.933751 0.000000 0.037463 -0.000000

2.931141 5.796024 0.481677 0.000000 -0.037463 -0.000000

0.135636	5.796024	2.226037	0.000000	-0.037463	0.000000
5.455373	1.932008	3.189392	-0.000000	0.037463	-0.000000
4.440201	0.366692	1.112822	-0.260663	-0.058133	-0.229669
1.150808	7.361340	4.302607	0.260663	0.058133	0.229669
3.946313	7.361340	3.820536	0.260663	0.058133	-0.229669
1.644697	0.366692	1.594893	-0.260663	-0.058133	0.229669
1.150808	4.230709	4.302607	-0.260663	0.058133	-0.229669
4.440201	3.497324	1.112822	0.260663	-0.058133	0.229669
1.644697	3.497324	1.594893	0.260663	-0.058133	-0.229669
3.946313	4.230709	3.820536	-0.260663	0.058133	0.229669

--

A_g(6); Fe-O2 stretching, in-phase

18 f = 12.556127 THz 78.892475 2PiTHz 418.827313 cm-1 51.927987 meV

X	Y	Z	dx	dy	dz
0.303633	1.932008	0.073534	-0.009979	-0.000000	-0.002003
5.287376	5.796024	5.341894	0.009979	0.000000	0.002003
2.491871	5.796024	2.781249	0.009979	-0.000000	-0.002003
3.099138	1.932008	2.634180	-0.009979	-0.000000	0.002003
0.000000	0.000000	2.707714	0.000000	0.000000	-0.000000
2.795505	0.000000	0.000000	0.000000	0.000000	0.000000
0.000000	3.864016	2.707714	0.000000	-0.000000	-0.000000
2.795505	3.864016	0.000000	-0.000000	-0.000000	0.000000
2.659869	1.932008	4.933751	-0.029100	-0.000000	-0.179308
2.931141	5.796024	0.481677	0.029100	0.000000	0.179308
0.135636	5.796024	2.226037	0.029100	-0.000000	-0.179308
5.455373	1.932008	3.189392	-0.029100	0.000000	0.179308
4.440201	0.366692	1.112822	0.212558	-0.111552	0.225443
1.150808	7.361340	4.302607	-0.212558	0.111552	-0.225443
3.946313	7.361340	3.820536	-0.212558	0.111552	0.225443

1.644697	0.366692	1.594893	0.212558	-0.111552	-0.225443
1.150808	4.230709	4.302607	-0.212558	-0.111552	-0.225443
4.440201	3.497324	1.112822	0.212558	0.111552	0.225443
1.644697	3.497324	1.594893	0.212558	0.111552	-0.225443
3.946313	4.230709	3.820536	-0.212558	-0.111552	0.225443

--

B_{2u}(7); O1 against Fe in x-y plane

19 f = 12.158088 THz 76.391520 2PiTHz 405.550153 cm-1 50.281829 meV

X	Y	Z	dx	dy	dz
0.303633	1.932008	0.073534	0.035728	0.000000	-0.030889
5.287376	5.796024	5.341894	0.035728	0.000000	-0.030889
2.491871	5.796024	2.781249	0.035728	-0.000000	0.030889
3.099138	1.932008	2.634180	0.035728	0.000000	0.030889
0.000000	0.000000	2.707714	0.018180	0.024541	0.196887
2.795505	0.000000	0.000000	0.018180	0.024541	-0.196887
0.000000	3.864016	2.707714	0.018180	-0.024541	0.196887
2.795505	3.864016	0.000000	0.018180	-0.024541	-0.196887
2.659869	1.932008	4.933751	-0.051624	0.000000	0.435821
2.931141	5.796024	0.481677	-0.051624	-0.000000	0.435821
0.135636	5.796024	2.226037	-0.051624	0.000000	-0.435821
5.455373	1.932008	3.189392	-0.051624	-0.000000	-0.435821
4.440201	0.366692	1.112822	-0.044661	0.037443	-0.065857
1.150808	7.361340	4.302607	-0.044661	0.037443	-0.065857
3.946313	7.361340	3.820536	-0.044661	0.037443	0.065857
1.644697	0.366692	1.594893	-0.044661	0.037443	0.065857
1.150808	4.230709	4.302607	-0.044661	-0.037443	-0.065857
4.440201	3.497324	1.112822	-0.044661	-0.037443	-0.065857
1.644697	3.497324	1.594893	-0.044661	-0.037443	0.065857
3.946313	4.230709	3.820536	-0.044661	-0.037443	0.065857

--

B_{3u}(7); O1-Fe-O2 scissor-like bending, Fe(xy)

20 f = 11.102204 THz 69.757206 2PiTHz 370.329655 cm-1 45.915042 meV

X	Y	Z	dx	dy	dz
0.303633	1.932008	0.073534	-0.004944	0.000000	-0.058448
5.287376	5.796024	5.341894	-0.004944	-0.000000	-0.058448
2.491871	5.796024	2.781249	0.004944	-0.000000	-0.058448
3.099138	1.932008	2.634180	0.004944	0.000000	-0.058448
0.000000	0.000000	2.707714	-0.168355	-0.035179	-0.135540
2.795505	0.000000	0.000000	0.168355	0.035179	-0.135540
0.000000	3.864016	2.707714	-0.168355	0.035179	-0.135540
2.795505	3.864016	0.000000	0.168355	-0.035179	-0.135540
2.659869	1.932008	4.933751	-0.132135	0.000000	0.162558
2.931141	5.796024	0.481677	-0.132135	-0.000000	0.162558
0.135636	5.796024	2.226037	0.132135	-0.000000	0.162558
5.455373	1.932008	3.189392	0.132135	0.000000	0.162558
4.440201	0.366692	1.112822	0.001871	-0.244480	0.132618
1.150808	7.361340	4.302607	0.001871	-0.244480	0.132618
3.946313	7.361340	3.820536	-0.001871	0.244480	0.132618
1.644697	0.366692	1.594893	-0.001871	0.244480	0.132618
1.150808	4.230709	4.302607	0.001871	0.244480	0.132618
4.440201	3.497324	1.112822	0.001871	0.244480	0.132618
1.644697	3.497324	1.594893	-0.001871	-0.244480	0.132618
3.946313	4.230709	3.820536	-0.001871	-0.244480	0.132618

--

A_g(5); $[110]_{pc}$ FeO_6 rotation, in-phase

21 f = 10.786891 THz 67.776032 2PiTHz 359.811929 cm-1 44.611010 meV

X	Y	Z	dx	dy	dz
0.303633	1.932008	0.073534	0.078842	-0.000000	0.015487

5.287376	5.796024	5.341894	-0.078842	-0.000000	-0.015487
2.491871	5.796024	2.781249	-0.078842	-0.000000	0.015487
3.099138	1.932008	2.634180	0.078842	0.000000	-0.015487
0.000000	0.000000	2.707714	-0.000000	-0.000000	-0.000000
2.795505	0.000000	0.000000	0.000000	-0.000000	-0.000000
0.000000	3.864016	2.707714	-0.000000	-0.000000	-0.000000
2.795505	3.864016	0.000000	0.000000	0.000000	-0.000000
2.659869	1.932008	4.933751	-0.224679	0.000000	0.230418
2.931141	5.796024	0.481677	0.224679	0.000000	-0.230418
0.135636	5.796024	2.226037	0.224679	-0.000000	0.230418
5.455373	1.932008	3.189392	-0.224679	0.000000	-0.230418
4.440201	0.366692	1.112822	-0.134438	-0.201751	0.105871
1.150808	7.361340	4.302607	0.134438	0.201751	-0.105871
3.946313	7.361340	3.820536	0.134438	0.201751	0.105871
1.644697	0.366692	1.594893	-0.134438	-0.201751	-0.105871
1.150808	4.230709	4.302607	0.134438	-0.201751	-0.105871
4.440201	3.497324	1.112822	-0.134438	0.201751	0.105871
1.644697	3.497324	1.594893	-0.134438	0.201751	-0.105871
3.946313	4.230709	3.820536	0.134438	-0.201751	0.105871

--

B_{3g}(3); $[001]_{pc}$ FeO_6 rotation, out-of-phase

22 f = 10.556274 THz 66.327028 2PiTHz 352.119398 cm-1 43.657257 meV

X	Y	Z	dx	dy	dz
0.303633	1.932008	0.073534	0.000000	-0.120377	-0.000000
5.287376	5.796024	5.341894	-0.000000	0.120377	-0.000000
2.491871	5.796024	2.781249	-0.000000	0.120377	-0.000000
3.099138	1.932008	2.634180	0.000000	-0.120377	0.000000
0.000000	0.000000	2.707714	0.000000	-0.000000	-0.000000
2.795505	0.000000	0.000000	0.000000	-0.000000	-0.000000

0.000000	3.864016	2.707714	-0.000000	0.000000	0.000000
2.795505	3.864016	0.000000	0.000000	0.000000	-0.000000
2.659869	1.932008	4.933751	-0.000000	-0.167925	0.000000
2.931141	5.796024	0.481677	0.000000	0.167925	0.000000
0.135636	5.796024	2.226037	0.000000	0.167925	0.000000
5.455373	1.932008	3.189392	0.000000	-0.167925	-0.000000
4.440201	0.366692	1.112822	0.036556	0.290340	-0.134244
1.150808	7.361340	4.302607	-0.036556	-0.290340	0.134244
3.946313	7.361340	3.820536	-0.036556	-0.290340	-0.134244
1.644697	0.366692	1.594893	0.036556	0.290340	0.134244
1.150808	4.230709	4.302607	0.036556	-0.290340	-0.134244
4.440201	3.497324	1.112822	-0.036556	0.290340	0.134244
1.644697	3.497324	1.594893	-0.036556	0.290340	-0.134244
3.946313	4.230709	3.820536	0.036556	-0.290340	0.134244

--

$B_{1g}(4)$; O1 x-y plane

23 f = 10.373701 THz 65.179887 2PiTHz 346.029415 cm-1 42.902195 meV

X	Y	Z	dx	dy	dz
0.303633	1.932008	0.073534	-0.001713	0.000000	0.066920
5.287376	5.796024	5.341894	0.001713	-0.000000	-0.066920
2.491871	5.796024	2.781249	-0.001713	-0.000000	-0.066920
3.099138	1.932008	2.634180	0.001713	-0.000000	0.066920
0.000000	0.000000	2.707714	0.000000	-0.000000	-0.000000
2.795505	0.000000	0.000000	-0.000000	0.000000	-0.000000
0.000000	3.864016	2.707714	0.000000	0.000000	-0.000000
2.795505	3.864016	0.000000	-0.000000	-0.000000	0.000000
2.659869	1.932008	4.933751	-0.192854	0.000000	-0.405874
2.931141	5.796024	0.481677	0.192854	-0.000000	0.405874
0.135636	5.796024	2.226037	-0.192854	-0.000000	0.405874

5.455373	1.932008	3.189392	0.192854	0.000000	-0.405874
4.440201	0.366692	1.112822	0.018045	-0.096994	-0.109832
1.150808	7.361340	4.302607	-0.018045	0.096994	0.109832
3.946313	7.361340	3.820536	0.018045	-0.096994	0.109832
1.644697	0.366692	1.594893	-0.018045	0.096994	-0.109832
1.150808	4.230709	4.302607	-0.018045	-0.096994	0.109832
4.440201	3.497324	1.112822	0.018045	0.096994	-0.109832
1.644697	3.497324	1.594893	-0.018045	-0.096994	-0.109832
3.946313	4.230709	3.820536	0.018045	0.096994	0.109832

--

B_{3u}(6); O1 x-y plane, Fe(yz)

24 f = 10.268419 THz 64.518380 2PiTHz 342.517583 cm-1 42.466783 meV

X	Y	Z	dx	dy	dz
0.303633	1.932008	0.073534	-0.137461	-0.000000	0.033455
5.287376	5.796024	5.341894	-0.137461	-0.000000	0.033455
2.491871	5.796024	2.781249	0.137461	0.000000	0.033455
3.099138	1.932008	2.634180	0.137461	0.000000	0.033455
0.000000	0.000000	2.707714	-0.288510	0.277108	0.046872
2.795505	0.000000	0.000000	0.288510	-0.277108	0.046872
0.000000	3.864016	2.707714	-0.288510	-0.277108	0.046872
2.795505	3.864016	0.000000	0.288510	0.277108	0.046872
2.659869	1.932008	4.933751	-0.101569	0.000000	-0.201316
2.931141	5.796024	0.481677	-0.101569	0.000000	-0.201316
0.135636	5.796024	2.226037	0.101569	-0.000000	-0.201316
5.455373	1.932008	3.189392	0.101569	-0.000000	-0.201316
4.440201	0.366692	1.112822	-0.087520	0.027399	0.006916
1.150808	7.361340	4.302607	-0.087520	0.027399	0.006916
3.946313	7.361340	3.820536	0.087520	-0.027399	0.006916
1.644697	0.366692	1.594893	0.087520	-0.027399	0.006916

1.150808	4.230709	4.302607	-0.087520	-0.027399	0.006916
4.440201	3.497324	1.112822	-0.087520	-0.027399	0.006916
1.644697	3.497324	1.594893	0.087520	0.027399	0.006916
3.946313	4.230709	3.820536	0.087520	0.027399	0.006916

--

A_u(6) silent mode

25 f = 10.255425 THz 64.436734 2PiTHz 342.084139 cm-1 42.413043 meV

X	Y	Z	dx	dy	dz
0.303633	1.932008	0.073534	-0.000000	0.086659	0.000000
5.287376	5.796024	5.341894	-0.000000	0.086659	0.000000
2.491871	5.796024	2.781249	0.000000	-0.086659	0.000000
3.099138	1.932008	2.634180	0.000000	-0.086659	0.000000
0.000000	0.000000	2.707714	-0.163783	0.019650	-0.156828
2.795505	0.000000	0.000000	0.163783	-0.019650	-0.156828
0.000000	3.864016	2.707714	0.163783	0.019650	0.156828
2.795505	3.864016	0.000000	-0.163783	-0.019650	0.156828
2.659869	1.932008	4.933751	-0.000000	-0.043430	-0.000000
2.931141	5.796024	0.481677	-0.000000	-0.043430	-0.000000
0.135636	5.796024	2.226037	0.000000	0.043430	-0.000000
5.455373	1.932008	3.189392	0.000000	0.043430	-0.000000
4.440201	0.366692	1.112822	-0.086842	-0.204571	0.212152
1.150808	7.361340	4.302607	-0.086842	-0.204571	0.212152
3.946313	7.361340	3.820536	0.086842	0.204571	0.212152
1.644697	0.366692	1.594893	0.086842	0.204571	0.212152
1.150808	4.230709	4.302607	0.086842	-0.204571	-0.212152
4.440201	3.497324	1.112822	0.086842	-0.204571	-0.212152
1.644697	3.497324	1.594893	-0.086842	0.204571	-0.212152
3.946313	4.230709	3.820536	-0.086842	0.204571	-0.212152

--

$B_{1u}(5)$; $[001]_{pc}$ FeO_6 rotation, out-of-phase, Fe(xyz)

26 f = 10.119678 THz 63.583814 2PiTHz 337.556125 cm-1 41.851641 meV

X	Y	Z	dx	dy	dz
0.303633	1.932008	0.073534	-0.000000	0.062040	-0.000000
5.287376	5.796024	5.341894	-0.000000	0.062040	0.000000
2.491871	5.796024	2.781249	0.000000	0.062040	0.000000
3.099138	1.932008	2.634180	0.000000	0.062040	0.000000
0.000000	0.000000	2.707714	0.121813	0.143299	-0.097769
2.795505	0.000000	0.000000	0.121813	0.143299	0.097769
0.000000	3.864016	2.707714	-0.121813	0.143299	0.097769
2.795505	3.864016	0.000000	-0.121813	0.143299	-0.097769
2.659869	1.932008	4.933751	0.000000	-0.109792	0.000000
2.931141	5.796024	0.481677	-0.000000	-0.109792	-0.000000
0.135636	5.796024	2.226037	0.000000	-0.109792	-0.000000
5.455373	1.932008	3.189392	0.000000	-0.109792	-0.000000
4.440201	0.366692	1.112822	-0.235171	-0.171430	0.099442
1.150808	7.361340	4.302607	-0.235171	-0.171430	0.099442
3.946313	7.361340	3.820536	-0.235171	-0.171430	-0.099442
1.644697	0.366692	1.594893	-0.235171	-0.171430	-0.099442
1.150808	4.230709	4.302607	0.235171	-0.171430	-0.099442
4.440201	3.497324	1.112822	0.235171	-0.171430	-0.099442
1.644697	3.497324	1.594893	0.235171	-0.171430	0.099442
3.946313	4.230709	3.820536	0.235171	-0.171430	0.099442

--

$B_{2u}(6)$; O2-Fe-O2 scissor-like bending, Fe(xz)

27 f = 9.656441 THz 60.673206 2PiTHz 322.104181 cm-1 39.935843 meV

X	Y	Z	dx	dy	dz
0.303633	1.932008	0.073534	-0.016776	-0.000000	0.172042
5.287376	5.796024	5.341894	-0.016776	0.000000	0.172042

2.491871	5.796024	2.781249	-0.016776	0.000000	-0.172042
3.099138	1.932008	2.634180	-0.016776	0.000000	-0.172042
0.000000	0.000000	2.707714	-0.019157	0.197209	-0.288543
2.795505	0.000000	0.000000	-0.019157	0.197209	0.288543
0.000000	3.864016	2.707714	-0.019157	-0.197209	-0.288543
2.795505	3.864016	0.000000	-0.019157	-0.197209	0.288543
2.659869	1.932008	4.933751	-0.155795	-0.000000	0.095504
2.931141	5.796024	0.481677	-0.155795	0.000000	0.095504
0.135636	5.796024	2.226037	-0.155795	0.000000	-0.095504
5.455373	1.932008	3.189392	-0.155795	-0.000000	-0.095504
4.440201	0.366692	1.112822	0.120782	0.033053	-0.128161
1.150808	7.361340	4.302607	0.120782	0.033053	-0.128161
3.946313	7.361340	3.820536	0.120782	0.033053	0.128161
1.644697	0.366692	1.594893	0.120782	0.033053	0.128161
1.150808	4.230709	4.302607	0.120782	-0.033053	-0.128161
4.440201	3.497324	1.112822	0.120782	-0.033053	-0.128161
1.644697	3.497324	1.594893	0.120782	-0.033053	0.128161
3.946313	4.230709	3.820536	0.120782	-0.033053	0.128161

--

B_{2g}(2); O1-Fe-O2 in-phase

28 f = 9.454635 THz 59.405224 2PiTHz 315.372671 cm-1 39.101242 meV

X	Y	Z	dx	dy	dz
0.303633	1.932008	0.073534	-0.000000	-0.073078	-0.000000
5.287376	5.796024	5.341894	-0.000000	0.073078	-0.000000
2.491871	5.796024	2.781249	0.000000	-0.073078	-0.000000
3.099138	1.932008	2.634180	0.000000	0.073078	-0.000000
0.000000	0.000000	2.707714	0.000000	0.000000	0.000000
2.795505	0.000000	0.000000	-0.000000	-0.000000	0.000000
0.000000	3.864016	2.707714	0.000000	-0.000000	0.000000

2.795505	3.864016	0.000000	0.000000	0.000000	-0.000000
2.659869	1.932008	4.933751	0.000000	-0.038065	0.000000
2.931141	5.796024	0.481677	0.000000	0.038065	0.000000
0.135636	5.796024	2.226037	-0.000000	-0.038065	0.000000
5.455373	1.932008	3.189392	-0.000000	0.038065	0.000000
4.440201	0.366692	1.112822	-0.087953	0.320810	0.104645
1.150808	7.361340	4.302607	0.087953	-0.320810	-0.104645
3.946313	7.361340	3.820536	-0.087953	0.320810	-0.104645
1.644697	0.366692	1.594893	0.087953	-0.320810	0.104645
1.150808	4.230709	4.302607	-0.087953	-0.320810	0.104645
4.440201	3.497324	1.112822	0.087953	0.320810	-0.104645
1.644697	3.497324	1.594893	-0.087953	-0.320810	-0.104645
3.946313	4.230709	3.820536	0.087953	0.320810	0.104645

--

B_{3u}(5); O1 x-y plane, out-of-phase, Fe(z)

29 f = 9.359596 THz 58.808076 2PiTHz 312.202505 cm-1 38.708191 meV

X	Y	Z	dx	dy	dz
0.303633	1.932008	0.073534	0.067388	-0.000000	0.029506
5.287376	5.796024	5.341894	0.067388	0.000000	0.029506
2.491871	5.796024	2.781249	-0.067388	-0.000000	0.029506
3.099138	1.932008	2.634180	-0.067388	0.000000	0.029506
0.000000	0.000000	2.707714	0.002408	-0.295749	-0.023115
2.795505	0.000000	0.000000	-0.002408	0.295749	-0.023115
0.000000	3.864016	2.707714	0.002408	0.295749	-0.023115
2.795505	3.864016	0.000000	-0.002408	-0.295749	-0.023115
2.659869	1.932008	4.933751	-0.260929	-0.000000	-0.197320
2.931141	5.796024	0.481677	-0.260929	0.000000	-0.197320
0.135636	5.796024	2.226037	0.260929	-0.000000	-0.197320
5.455373	1.932008	3.189392	0.260929	0.000000	-0.197320

4.440201	0.366692	1.112822	-0.120061	0.067829	0.075928
1.150808	7.361340	4.302607	-0.120061	0.067829	0.075928
3.946313	7.361340	3.820536	0.120061	-0.067829	0.075928
1.644697	0.366692	1.594893	0.120061	-0.067829	0.075928
1.150808	4.230709	4.302607	-0.120061	-0.067829	0.075928
4.440201	3.497324	1.112822	-0.120061	-0.067829	0.075928
1.644697	3.497324	1.594893	0.120061	0.067829	0.075928
3.946313	4.230709	3.820536	0.120061	0.067829	0.075928

--

A_g(4); O1 x-y plane

30 f = 9.218138 THz 57.919268 2PiTHz 307.483972 cm-1 38.123167 meV

X	Y	Z	dx	dy	dz
0.303633	1.932008	0.073534	-0.015356	0.000000	0.010561
5.287376	5.796024	5.341894	0.015356	0.000000	-0.010561
2.491871	5.796024	2.781249	0.015356	0.000000	0.010561
3.099138	1.932008	2.634180	-0.015356	0.000000	-0.010561
0.000000	0.000000	2.707714	0.000000	-0.000000	0.000000
2.795505	0.000000	0.000000	-0.000000	0.000000	-0.000000
0.000000	3.864016	2.707714	-0.000000	-0.000000	-0.000000
2.795505	3.864016	0.000000	0.000000	-0.000000	0.000000
2.659869	1.932008	4.933751	0.431088	-0.000000	0.183488
2.931141	5.796024	0.481677	-0.431088	0.000000	-0.183488
0.135636	5.796024	2.226037	-0.431088	-0.000000	0.183488
5.455373	1.932008	3.189392	0.431088	-0.000000	-0.183488
4.440201	0.366692	1.112822	-0.032630	-0.063795	0.099697
1.150808	7.361340	4.302607	0.032630	0.063795	-0.099697
3.946313	7.361340	3.820536	0.032630	0.063795	0.099697
1.644697	0.366692	1.594893	-0.032630	-0.063795	-0.099697
1.150808	4.230709	4.302607	0.032630	-0.063795	-0.099697

4.440201	3.497324	1.112822	-0.032630	0.063795	0.099697
1.644697	3.497324	1.594893	-0.032630	0.063795	-0.099697
3.946313	4.230709	3.820536	0.032630	-0.063795	0.099697

--

A_u(5) silent mode

31 f = 9.095308 THz 57.147506 2PiTHz 303.386811 cm-1 37.615184 meV

X	Y	Z	dx	dy	dz
0.303633	1.932008	0.073534	-0.000000	0.025431	-0.000000
5.287376	5.796024	5.341894	0.000000	0.025431	-0.000000
2.491871	5.796024	2.781249	-0.000000	-0.025431	0.000000
3.099138	1.932008	2.634180	-0.000000	-0.025431	0.000000
0.000000	0.000000	2.707714	-0.432411	0.075469	-0.032918
2.795505	0.000000	0.000000	0.432411	-0.075469	-0.032918
0.000000	3.864016	2.707714	0.432411	0.075469	0.032918
2.795505	3.864016	0.000000	-0.432411	-0.075469	0.032918
2.659869	1.932008	4.933751	0.000000	-0.017193	-0.000000
2.931141	5.796024	0.481677	-0.000000	-0.017193	-0.000000
0.135636	5.796024	2.226037	-0.000000	0.017193	0.000000
5.455373	1.932008	3.189392	0.000000	0.017193	0.000000
4.440201	0.366692	1.112822	-0.019451	0.159168	-0.044011
1.150808	7.361340	4.302607	-0.019451	0.159168	-0.044011
3.946313	7.361340	3.820536	0.019451	-0.159168	-0.044011
1.644697	0.366692	1.594893	0.019451	-0.159168	-0.044011
1.150808	4.230709	4.302607	0.019451	0.159168	0.044011
4.440201	3.497324	1.112822	0.019451	0.159168	0.044011
1.644697	3.497324	1.594893	-0.019451	-0.159168	0.044011
3.946313	4.230709	3.820536	-0.019451	-0.159168	0.044011

--

B_{2u}(5); O2-Fe-O2 scissor-like bending, Fe(xyz)

32 f =   9.065002 THz   56.957087 2PiTHz 302.375906 cm-1   37.489848 meV

X	Y	Z	dx	dy	dz
0.303633	1.932008	0.073534	-0.005398	-0.000000	-0.105795
5.287376	5.796024	5.341894	-0.005398	-0.000000	-0.105795
2.491871	5.796024	2.781249	-0.005398	0.000000	0.105795
3.099138	1.932008	2.634180	-0.005398	0.000000	0.105795
0.000000	0.000000	2.707714	-0.216414	0.227764	0.234349
2.795505	0.000000	0.000000	-0.216414	0.227764	-0.234349
0.000000	3.864016	2.707714	-0.216414	-0.227764	0.234349
2.795505	3.864016	0.000000	-0.216414	-0.227764	-0.234349
2.659869	1.932008	4.933751	0.092279	0.000000	-0.100890
2.931141	5.796024	0.481677	0.092279	0.000000	-0.100890
0.135636	5.796024	2.226037	0.092279	-0.000000	0.100890
5.455373	1.932008	3.189392	0.092279	-0.000000	0.100890
4.440201	0.366692	1.112822	0.162743	0.034719	-0.074403
1.150808	7.361340	4.302607	0.162743	0.034719	-0.074403
3.946313	7.361340	3.820536	0.162743	0.034719	0.074403
1.644697	0.366692	1.594893	0.162743	0.034719	0.074403
1.150808	4.230709	4.302607	0.162743	-0.034719	-0.074403
4.440201	3.497324	1.112822	0.162743	-0.034719	-0.074403
1.644697	3.497324	1.594893	0.162743	-0.034719	0.074403
3.946313	4.230709	3.820536	0.162743	-0.034719	0.074403

--

B_{1u}(4); Fe(x) out-of-phase in z

33 f =   9.043366 THz   56.821142 2PiTHz 301.654197 cm-1   37.400367 meV

X	Y	Z	dx	dy	dz
0.303633	1.932008	0.073534	0.000000	0.083575	0.000000
5.287376	5.796024	5.341894	-0.000000	0.083575	0.000000
2.491871	5.796024	2.781249	-0.000000	0.083575	-0.000000

3.099138	1.932008	2.634180	0.000000	0.083575	-0.000000
0.000000	0.000000	2.707714	0.059395	-0.149746	0.432326
2.795505	0.000000	0.000000	0.059395	-0.149746	-0.432326
0.000000	3.864016	2.707714	-0.059395	-0.149746	-0.432326
2.795505	3.864016	0.000000	-0.059395	-0.149746	0.432326
2.659869	1.932008	4.933751	-0.000000	-0.029673	-0.000000
2.931141	5.796024	0.481677	0.000000	-0.029673	0.000000
0.135636	5.796024	2.226037	0.000000	-0.029673	-0.000000
5.455373	1.932008	3.189392	-0.000000	-0.029673	0.000000
4.440201	0.366692	1.112822	-0.092451	0.028772	0.072552
1.150808	7.361340	4.302607	-0.092451	0.028772	0.072552
3.946313	7.361340	3.820536	-0.092451	0.028772	-0.072552
1.644697	0.366692	1.594893	-0.092451	0.028772	-0.072552
1.150808	4.230709	4.302607	0.092451	0.028772	-0.072552
4.440201	3.497324	1.112822	0.092451	0.028772	-0.072552
1.644697	3.497324	1.594893	0.092451	0.028772	0.072552
3.946313	4.230709	3.820536	0.092451	0.028772	0.072552

--

B_{2u}(4); O1 against Fe x-y plane, in-phase

34 f = 8.465199 THz 53.188412 2PiTHz 282.368624 cm-1 35.009260 meV

X	Y	Z	dx	dy	dz
0.303633	1.932008	0.073534	0.049082	0.000000	-0.086725
5.287376	5.796024	5.341894	0.049082	0.000000	-0.086725
2.491871	5.796024	2.781249	0.049082	0.000000	0.086725
3.099138	1.932008	2.634180	0.049082	-0.000000	0.086725
0.000000	0.000000	2.707714	0.128562	-0.020390	0.154187
2.795505	0.000000	0.000000	0.128562	-0.020390	-0.154187
0.000000	3.864016	2.707714	0.128562	0.020390	0.154187
2.795505	3.864016	0.000000	0.128562	0.020390	-0.154187

2.659869	1.932008	4.933751	-0.404712	0.000000	-0.143023
2.931141	5.796024	0.481677	-0.404712	0.000000	-0.143023
0.135636	5.796024	2.226037	-0.404712	0.000000	0.143023
5.455373	1.932008	3.189392	-0.404712	0.000000	0.143023
4.440201	0.366692	1.112822	0.009751	-0.049075	-0.071056
1.150808	7.361340	4.302607	0.009751	-0.049075	-0.071056
3.946313	7.361340	3.820536	0.009751	-0.049075	0.071056
1.644697	0.366692	1.594893	0.009751	-0.049075	0.071056
1.150808	4.230709	4.302607	0.009751	0.049075	-0.071056
4.440201	3.497324	1.112822	0.009751	0.049075	-0.071056
1.644697	3.497324	1.594893	0.009751	0.049075	0.071056
3.946313	4.230709	3.820536	0.009751	0.049075	0.071056

--

B_{3u}(4); O1-Fe-O2 scissor-like bending, Fe(xyz)

35 f = 8.314639 THz 52.242421 2PiTHz 277.346511 cm-1 34.386597 meV

X	Y	Z	dx	dy	dz
0.303633	1.932008	0.073534	-0.135151	-0.000000	0.061922
5.287376	5.796024	5.341894	-0.135151	0.000000	0.061922
2.491871	5.796024	2.781249	0.135151	-0.000000	0.061922
3.099138	1.932008	2.634180	0.135151	0.000000	0.061922
0.000000	0.000000	2.707714	-0.232033	-0.176756	-0.194835
2.795505	0.000000	0.000000	0.232033	0.176756	-0.194835
0.000000	3.864016	2.707714	-0.232033	0.176756	-0.194835
2.795505	3.864016	0.000000	0.232033	-0.176756	-0.194835
2.659869	1.932008	4.933751	0.239708	-0.000000	0.091877
2.931141	5.796024	0.481677	0.239708	-0.000000	0.091877
0.135636	5.796024	2.226037	-0.239708	-0.000000	0.091877
5.455373	1.932008	3.189392	-0.239708	-0.000000	0.091877
4.440201	0.366692	1.112822	-0.032631	0.128975	0.042171

1.150808	7.361340	4.302607	-0.032631	0.128975	0.042171
3.946313	7.361340	3.820536	0.032631	-0.128975	0.042171
1.644697	0.366692	1.594893	0.032631	-0.128975	0.042171
1.150808	4.230709	4.302607	-0.032631	-0.128975	0.042171
4.440201	3.497324	1.112822	-0.032631	-0.128975	0.042171
1.644697	3.497324	1.594893	0.032631	0.128975	0.042171
3.946313	4.230709	3.820536	0.032631	0.128975	0.042171

--

B_{1u}(3); O2(z) , O2(z) out-of-phase in z, in-phase in x-y Fe(xyz)

36 f = 8.147969 THz 51.195198 2PiTHz 271.786974 cm-1 33.697302 meV

X	Y	Z	dx	dy	dz
0.303633	1.932008	0.073534	-0.000000	0.019631	0.000000
5.287376	5.796024	5.341894	-0.000000	0.019631	0.000000
2.491871	5.796024	2.781249	0.000000	0.019631	0.000000
3.099138	1.932008	2.634180	0.000000	0.019631	0.000000
0.000000	0.000000	2.707714	0.196128	-0.254465	-0.208451
2.795505	0.000000	0.000000	0.196128	-0.254465	0.208451
0.000000	3.864016	2.707714	-0.196128	-0.254465	0.208451
2.795505	3.864016	0.000000	-0.196128	-0.254465	-0.208451
2.659869	1.932008	4.933751	0.000000	0.036130	0.000000
2.931141	5.796024	0.481677	0.000000	0.036130	0.000000
0.135636	5.796024	2.226037	0.000000	0.036130	0.000000
5.455373	1.932008	3.189392	-0.000000	0.036130	-0.000000
4.440201	0.366692	1.112822	-0.092060	0.188679	0.082125
1.150808	7.361340	4.302607	-0.092060	0.188679	0.082125
3.946313	7.361340	3.820536	-0.092060	0.188679	-0.082125
1.644697	0.366692	1.594893	-0.092060	0.188679	-0.082125
1.150808	4.230709	4.302607	0.092060	0.188679	-0.082125
4.440201	3.497324	1.112822	0.092060	0.188679	-0.082125

1.644697	3.497324	1.594893	0.092060	0.188679	0.082125
3.946313	4.230709	3.820536	0.092060	0.188679	0.082125

--

B_{3u}(3); $[110]_{pc}$ FeO_6 rotation in-phase, Fe(xyz)

37 f = 7.687662 THz 48.303007 2PiTHz 256.432804 cm-1 31.793627 meV

X	Y	Z	dx	dy	dz
0.303633	1.932008	0.073534	0.070560	0.000000	-0.000368
5.287376	5.796024	5.341894	0.070560	0.000000	-0.000368
2.491871	5.796024	2.781249	-0.070560	-0.000000	-0.000368
3.099138	1.932008	2.634180	-0.070560	0.000000	-0.000368
0.000000	0.000000	2.707714	0.182231	0.225542	-0.227052
2.795505	0.000000	0.000000	-0.182231	-0.225542	-0.227052
0.000000	3.864016	2.707714	0.182231	-0.225542	-0.227052
2.795505	3.864016	0.000000	-0.182231	0.225542	-0.227052
2.659869	1.932008	4.933751	-0.046905	0.000000	0.097354
2.931141	5.796024	0.481677	-0.046905	0.000000	0.097354
0.135636	5.796024	2.226037	0.046905	-0.000000	0.097354
5.455373	1.932008	3.189392	0.046905	-0.000000	0.097354
4.440201	0.366692	1.112822	-0.095270	0.115728	0.162443
1.150808	7.361340	4.302607	-0.095270	0.115728	0.162443
3.946313	7.361340	3.820536	0.095270	-0.115728	0.162443
1.644697	0.366692	1.594893	0.095270	-0.115728	0.162443
1.150808	4.230709	4.302607	-0.095270	-0.115728	0.162443
4.440201	3.497324	1.112822	-0.095270	-0.115728	0.162443
1.644697	3.497324	1.594893	0.095270	0.115728	0.162443
3.946313	4.230709	3.820536	0.095270	0.115728	0.162443

--

B_{1g}(3); $[110]_{pc}$ FeO_6 rotation, in-phase

38 f = 7.552157 THz 47.451599 2PiTHz 251.912818 cm-1 31.233220 meV

X	Y	Z	dx	dy	dz
0.303633	1.932008	0.073534	0.055237	0.000000	-0.003172
5.287376	5.796024	5.341894	-0.055237	0.000000	0.003172
2.491871	5.796024	2.781249	0.055237	-0.000000	0.003172
3.099138	1.932008	2.634180	-0.055237	-0.000000	-0.003172
0.000000	0.000000	2.707714	0.000000	-0.000000	-0.000000
2.795505	0.000000	0.000000	-0.000000	0.000000	-0.000000
0.000000	3.864016	2.707714	-0.000000	-0.000000	0.000000
2.795505	3.864016	0.000000	0.000000	0.000000	0.000000
2.659869	1.932008	4.933751	-0.317387	0.000000	-0.000238
2.931141	5.796024	0.481677	0.317387	0.000000	0.000238
0.135636	5.796024	2.226037	-0.317387	-0.000000	0.000238
5.455373	1.932008	3.189392	0.317387	-0.000000	-0.000238
4.440201	0.366692	1.112822	-0.049046	0.263283	0.037127
1.150808	7.361340	4.302607	0.049046	-0.263283	-0.037127
3.946313	7.361340	3.820536	-0.049046	0.263283	-0.037127
1.644697	0.366692	1.594893	0.049046	-0.263283	0.037127
1.150808	4.230709	4.302607	0.049046	0.263283	-0.037127
4.440201	3.497324	1.112822	-0.049046	-0.263283	0.037127
1.644697	3.497324	1.594893	0.049046	0.263283	0.037127
3.946313	4.230709	3.820536	-0.049046	-0.263283	-0.037127

--

A_u(4) silent mode

39 f = 7.551418 THz 47.446960 2PiTHz 251.888191 cm-1 31.230167 meV

X	Y	Z	dx	dy	dz
0.303633	1.932008	0.073534	0.000000	-0.007826	-0.000000
5.287376	5.796024	5.341894	-0.000000	-0.007826	0.000000
2.491871	5.796024	2.781249	0.000000	0.007826	0.000000
3.099138	1.932008	2.634180	-0.000000	0.007826	-0.000000

0.000000	0.000000	2.707714	-0.146145	0.088431	0.206216
2.795505	0.000000	0.000000	0.146145	-0.088431	0.206216
0.000000	3.864016	2.707714	0.146145	0.088431	-0.206216
2.795505	3.864016	0.000000	-0.146145	-0.088431	-0.206216
2.659869	1.932008	4.933751	-0.000000	-0.008365	0.000000
2.931141	5.796024	0.481677	0.000000	-0.008365	0.000000
0.135636	5.796024	2.226037	-0.000000	0.008365	0.000000
5.455373	1.932008	3.189392	0.000000	0.008365	0.000000
4.440201	0.366692	1.112822	0.112842	-0.227540	-0.156764
1.150808	7.361340	4.302607	0.112842	-0.227540	-0.156764
3.946313	7.361340	3.820536	-0.112842	0.227540	-0.156764
1.644697	0.366692	1.594893	-0.112842	0.227540	-0.156764
1.150808	4.230709	4.302607	-0.112842	-0.227540	0.156764
4.440201	3.497324	1.112822	-0.112842	-0.227540	0.156764
1.644697	3.497324	1.594893	0.112842	0.227540	0.156764
3.946313	4.230709	3.820536	0.112842	0.227540	0.156764

--

B_{2u}(3); O1-Fe-O2 in-phase, Fe(y)

40 f = 7.280820 THz 45.746738 2PiTHz 242.861990 cm-1 30.111060 meV

X	Y	Z	dx	dy	dz
0.303633	1.932008	0.073534	0.039750	-0.000000	-0.031603
5.287376	5.796024	5.341894	0.039750	-0.000000	-0.031603
2.491871	5.796024	2.781249	0.039750	0.000000	0.031603
3.099138	1.932008	2.634180	0.039750	0.000000	0.031603
0.000000	0.000000	2.707714	0.122368	0.330064	0.001458
2.795505	0.000000	0.000000	0.122368	0.330064	-0.001458
0.000000	3.864016	2.707714	0.122368	-0.330064	0.001458
2.795505	3.864016	0.000000	0.122368	-0.330064	-0.001458
2.659869	1.932008	4.933751	-0.023275	0.000000	-0.062517

2.931141	5.796024	0.481677	-0.023275	0.000000	-0.062517
0.135636	5.796024	2.226037	-0.023275	-0.000000	0.062517
5.455373	1.932008	3.189392	-0.023275	-0.000000	0.062517
4.440201	0.366692	1.112822	-0.161134	0.154644	0.098223
1.150808	7.361340	4.302607	-0.161134	0.154644	0.098223
3.946313	7.361340	3.820536	-0.161134	0.154644	-0.098223
1.644697	0.366692	1.594893	-0.161134	0.154644	-0.098223
1.150808	4.230709	4.302607	-0.161134	-0.154644	0.098223
4.440201	3.497324	1.112822	-0.161134	-0.154644	0.098223
1.644697	3.497324	1.594893	-0.161134	-0.154644	-0.098223
3.946313	4.230709	3.820536	-0.161134	-0.154644	-0.098223

--

A_g(3); $[001]_{pc}$ FeO_6 rotation, in-phase

41 f = 6.940679 THz 43.609573 2PiTHz 231.516128 cm-1 28.704352 meV

X	Y	Z	dx	dy	dz
0.303633	1.932008	0.073534	-0.111794	0.000000	0.162905
5.287376	5.796024	5.341894	0.111794	-0.000000	-0.162905
2.491871	5.796024	2.781249	0.111794	-0.000000	0.162905
3.099138	1.932008	2.634180	-0.111794	0.000000	-0.162905
0.000000	0.000000	2.707714	-0.000000	-0.000000	0.000000
2.795505	0.000000	0.000000	-0.000000	-0.000000	-0.000000
0.000000	3.864016	2.707714	-0.000000	0.000000	0.000000
2.795505	3.864016	0.000000	0.000000	0.000000	0.000000
2.659869	1.932008	4.933751	0.004023	-0.000000	0.135286
2.931141	5.796024	0.481677	-0.004023	0.000000	-0.135286
0.135636	5.796024	2.226037	-0.004023	0.000000	0.135286
5.455373	1.932008	3.189392	0.004023	0.000000	-0.135286
4.440201	0.366692	1.112822	0.178127	-0.162026	-0.195809
1.150808	7.361340	4.302607	-0.178127	0.162026	0.195809

3.946313	7.361340	3.820536	-0.178127	0.162026	-0.195809
1.644697	0.366692	1.594893	0.178127	-0.162026	0.195809
1.150808	4.230709	4.302607	-0.178127	-0.162026	0.195809
4.440201	3.497324	1.112822	0.178127	0.162026	-0.195809
1.644697	3.497324	1.594893	0.178127	0.162026	0.195809
3.946313	4.230709	3.820536	-0.178127	-0.162026	-0.195809

--

$B_{3g}(2)$; $[001]_{pc}$ FeO_6 rotation, out-of-phase

42 f = 6.436719 THz 40.443101 2PiTHz 214.705842 cm-1 26.620141 meV

X	Y	Z	dx	dy	dz
0.303633	1.932008	0.073534	-0.000000	0.090907	-0.000000
5.287376	5.796024	5.341894	-0.000000	-0.090907	0.000000
2.491871	5.796024	2.781249	-0.000000	-0.090907	0.000000
3.099138	1.932008	2.634180	0.000000	0.090907	0.000000
0.000000	0.000000	2.707714	-0.000000	0.000000	-0.000000
2.795505	0.000000	0.000000	-0.000000	-0.000000	-0.000000
0.000000	3.864016	2.707714	-0.000000	0.000000	0.000000
2.795505	3.864016	0.000000	0.000000	-0.000000	-0.000000
2.659869	1.932008	4.933751	-0.000000	-0.002723	-0.000000
2.931141	5.796024	0.481677	-0.000000	0.002723	-0.000000
0.135636	5.796024	2.226037	-0.000000	0.002723	-0.000000
5.455373	1.932008	3.189392	-0.000000	-0.002723	-0.000000
4.440201	0.366692	1.112822	0.232517	-0.117627	-0.230139
1.150808	7.361340	4.302607	-0.232517	0.117627	0.230139
3.946313	7.361340	3.820536	-0.232517	0.117627	-0.230139
1.644697	0.366692	1.594893	0.232517	-0.117627	0.230139
1.150808	4.230709	4.302607	0.232517	0.117627	-0.230139
4.440201	3.497324	1.112822	-0.232517	-0.117627	0.230139
1.644697	3.497324	1.594893	-0.232517	-0.117627	-0.230139

3.946313 4.230709 3.820536   0.232517   0.117627   0.230139

--

A_u(3) silent mode

43 f =   6.008509 THz   37.752573 2PiTHz 200.422270 cm-1   24.849203 meV

X	Y	Z	dx	dy	dz
0.303633	1.932008	0.073534	0.000000	-0.042195	-0.000000
5.287376	5.796024	5.341894	0.000000	-0.042195	-0.000000
2.491871	5.796024	2.781249	0.000000	0.042195	-0.000000
3.099138	1.932008	2.634180	0.000000	0.042195	0.000000
0.000000	0.000000	2.707714	0.105326	0.448363	-0.145454
2.795505	0.000000	0.000000	-0.105326	-0.448363	-0.145454
0.000000	3.864016	2.707714	-0.105326	0.448363	0.145454
2.795505	3.864016	0.000000	0.105326	-0.448363	0.145454
2.659869	1.932008	4.933751	-0.000000	-0.105076	0.000000
2.931141	5.796024	0.481677	0.000000	-0.105076	0.000000
0.135636	5.796024	2.226037	-0.000000	0.105076	0.000000
5.455373	1.932008	3.189392	-0.000000	0.105076	0.000000
4.440201	0.366692	1.112822	-0.020752	0.006258	-0.038467
1.150808	7.361340	4.302607	-0.020752	0.006258	-0.038467
3.946313	7.361340	3.820536	0.020752	-0.006258	-0.038467
1.644697	0.366692	1.594893	0.020752	-0.006258	-0.038467
1.150808	4.230709	4.302607	0.020752	0.006258	0.038467
4.440201	3.497324	1.112822	0.020752	0.006258	0.038467
1.644697	3.497324	1.594893	-0.020752	-0.006258	0.038467
3.946313	4.230709	3.820536	-0.020752	-0.006258	0.038467

--

B_{2u}(2); Nd(y) against FeO_6 octahedra

44 f =   5.676322 THz   35.665381 2PiTHz 189.341707 cm-1   23.475388 meV

X   Y   Z   dx   dy   dz

0.303633	1.932008	0.073534	0.309948	0.000000	-0.027573
5.287376	5.796024	5.341894	0.309948	0.000000	-0.027573
2.491871	5.796024	2.781249	0.309948	0.000000	0.027573
3.099138	1.932008	2.634180	0.309948	0.000000	0.027573
0.000000	0.000000	2.707714	-0.328172	-0.049029	-0.083718
2.795505	0.000000	0.000000	-0.328172	-0.049029	0.083718
0.000000	3.864016	2.707714	-0.328172	0.049029	-0.083718
2.795505	3.864016	0.000000	-0.328172	0.049029	0.083718
2.659869	1.932008	4.933751	-0.090628	-0.000000	-0.014282
2.931141	5.796024	0.481677	-0.090628	-0.000000	-0.014282
0.135636	5.796024	2.226037	-0.090628	-0.000000	0.014282
5.455373	1.932008	3.189392	-0.090628	-0.000000	0.014282
4.440201	0.366692	1.112822	-0.115582	0.017620	-0.012367
1.150808	7.361340	4.302607	-0.115582	0.017620	-0.012367
3.946313	7.361340	3.820536	-0.115582	0.017620	0.012367
1.644697	0.366692	1.594893	-0.115582	0.017620	0.012367
1.150808	4.230709	4.302607	-0.115582	-0.017620	-0.012367
4.440201	3.497324	1.112822	-0.115582	-0.017620	-0.012367
1.644697	3.497324	1.594893	-0.115582	-0.017620	0.012367
3.946313	4.230709	3.820536	-0.115582	-0.017620	0.012367

--

B_{3u}(2); Nd(x) against FeO_6 octahedra

45 f = 5.311471 THz 33.372954 2PiTHz 177.171585 cm-1 21.966485 meV

X	Y	Z	dx	dy	dz
0.303633	1.932008	0.073534	-0.010453	0.000000	-0.307634
5.287376	5.796024	5.341894	-0.010453	0.000000	-0.307634
2.491871	5.796024	2.781249	0.010453	0.000000	-0.307634
3.099138	1.932008	2.634180	0.010453	0.000000	-0.307634
0.000000	0.000000	2.707714	-0.054586	-0.024808	0.279617

2.795505	0.000000	0.000000	0.054586	0.024808	0.279617
0.000000	3.864016	2.707714	-0.054586	0.024808	0.279617
2.795505	3.864016	0.000000	0.054586	-0.024808	0.279617
2.659869	1.932008	4.933751	0.047639	-0.000000	0.089181
2.931141	5.796024	0.481677	0.047639	-0.000000	0.089181
0.135636	5.796024	2.226037	-0.047639	-0.000000	0.089181
5.455373	1.932008	3.189392	-0.047639	-0.000000	0.089181
4.440201	0.366692	1.112822	-0.017231	0.081748	0.156986
1.150808	7.361340	4.302607	-0.017231	0.081748	0.156986
3.946313	7.361340	3.820536	0.017231	-0.081748	0.156986
1.644697	0.366692	1.594893	0.017231	-0.081748	0.156986
1.150808	4.230709	4.302607	-0.017231	-0.081748	0.156986
4.440201	3.497324	1.112822	-0.017231	-0.081748	0.156986
1.644697	3.497324	1.594893	0.017231	0.081748	0.156986
3.946313	4.230709	3.820536	0.017231	0.081748	0.156986

--

B_{1u}(2); Nd(z), in-phase in x, Fe+O1 y-z plane

46 f = 5.287595 THz 33.222937 2PiTHz 176.375166 cm-1 21.867741 meV

X	Y	Z	dx	dy	dz
0.303633	1.932008	0.073534	0.000000	-0.250303	-0.000000
5.287376	5.796024	5.341894	0.000000	-0.250303	-0.000000
2.491871	5.796024	2.781249	0.000000	-0.250303	-0.000000
3.099138	1.932008	2.634180	0.000000	-0.250303	-0.000000
0.000000	0.000000	2.707714	-0.202378	0.177683	0.041559
2.795505	0.000000	0.000000	-0.202378	0.177683	-0.041559
0.000000	3.864016	2.707714	0.202378	0.177683	-0.041559
2.795505	3.864016	0.000000	0.202378	0.177683	0.041559
2.659869	1.932008	4.933751	0.000000	0.118517	0.000000
2.931141	5.796024	0.481677	0.000000	0.118517	0.000000

0.135636	5.796024	2.226037	-0.000000	0.118517	0.000000
5.455373	1.932008	3.189392	-0.000000	0.118517	0.000000
4.440201	0.366692	1.112822	-0.154588	0.150683	0.054043
1.150808	7.361340	4.302607	-0.154588	0.150683	0.054043
3.946313	7.361340	3.820536	-0.154588	0.150683	-0.054043
1.644697	0.366692	1.594893	-0.154588	0.150683	-0.054043
1.150808	4.230709	4.302607	0.154588	0.150683	-0.054043
4.440201	3.497324	1.112822	0.154588	0.150683	-0.054043
1.644697	3.497324	1.594893	0.154588	0.150683	0.054043
3.946313	4.230709	3.820536	0.154588	0.150683	0.054043

--

A_u(2) silent mode

47 f = 5.045319 THz 31.700676 2PiTHz 168.293728 cm-1 20.865771 meV

X	Y	Z	dx	dy	dz
0.303633	1.932008	0.073534	-0.000000	0.157827	0.000000
5.287376	5.796024	5.341894	0.000000	0.157827	0.000000
2.491871	5.796024	2.781249	-0.000000	-0.157827	0.000000
3.099138	1.932008	2.634180	0.000000	-0.157827	0.000000
0.000000	0.000000	2.707714	0.026437	0.136798	0.375594
2.795505	0.000000	0.000000	-0.026437	-0.136798	0.375594
0.000000	3.864016	2.707714	-0.026437	0.136798	-0.375594
2.795505	3.864016	0.000000	0.026437	-0.136798	-0.375594
2.659869	1.932008	4.933751	-0.000000	-0.070936	-0.000000
2.931141	5.796024	0.481677	-0.000000	-0.070936	-0.000000
0.135636	5.796024	2.226037	0.000000	0.070936	-0.000000
5.455373	1.932008	3.189392	0.000000	0.070936	-0.000000
4.440201	0.366692	1.112822	-0.024061	0.066474	0.157448
1.150808	7.361340	4.302607	-0.024061	0.066474	0.157448
3.946313	7.361340	3.820536	0.024061	-0.066474	0.157448

1.644697	0.366692	1.594893	0.024061	-0.066474	0.157448
1.150808	4.230709	4.302607	0.024061	0.066474	-0.157448
4.440201	3.497324	1.112822	0.024061	0.066474	-0.157448
1.644697	3.497324	1.594893	-0.024061	-0.066474	-0.157448
3.946313	4.230709	3.820536	-0.024061	-0.066474	-0.157448

--

B_{1u}(1); Nd(z), Fe(y) out-of-phase in z, in-phase in x-y

48 f = 5.016359 THz 31.518710 2PiTHz 167.327705 cm-1 20.745999 meV

X	Y	Z	dx	dy	dz
0.303633	1.932008	0.073534	-0.000000	-0.174375	0.000000
5.287376	5.796024	5.341894	-0.000000	-0.174375	0.000000
2.491871	5.796024	2.781249	-0.000000	-0.174375	0.000000
3.099138	1.932008	2.634180	-0.000000	-0.174375	-0.000000
0.000000	0.000000	2.707714	0.378649	0.211715	0.087323
2.795505	0.000000	0.000000	0.378649	0.211715	-0.087323
0.000000	3.864016	2.707714	-0.378649	0.211715	-0.087323
2.795505	3.864016	0.000000	-0.378649	0.211715	0.087323
2.659869	1.932008	4.933751	0.000000	0.066136	-0.000000
2.931141	5.796024	0.481677	-0.000000	0.066136	-0.000000
0.135636	5.796024	2.226037	0.000000	0.066136	0.000000
5.455373	1.932008	3.189392	-0.000000	0.066136	-0.000000
4.440201	0.366692	1.112822	0.090771	0.032499	0.020062
1.150808	7.361340	4.302607	0.090771	0.032499	0.020062
3.946313	7.361340	3.820536	0.090771	0.032499	-0.020062
1.644697	0.366692	1.594893	0.090771	0.032499	-0.020062
1.150808	4.230709	4.302607	-0.090771	0.032499	-0.020062
4.440201	3.497324	1.112822	-0.090771	0.032499	-0.020062
1.644697	3.497324	1.594893	-0.090771	0.032499	0.020062
3.946313	4.230709	3.820536	-0.090771	0.032499	0.020062

--

B_{3g}(1); Nd(z) in-phase in x-y, out-of-phase in z

49 f = 4.817770 THz 30.270940 2PiTHz 160.703494 cm-1 19.924701 meV

X	Y	Z	dx	dy	dz
0.303633	1.932008	0.073534	0.000000	-0.470869	-0.000000
5.287376	5.796024	5.341894	0.000000	0.470869	-0.000000
2.491871	5.796024	2.781249	0.000000	0.470869	0.000000
3.099138	1.932008	2.634180	-0.000000	-0.470869	-0.000000
0.000000	0.000000	2.707714	-0.000000	0.000000	0.000000
2.795505	0.000000	0.000000	0.000000	-0.000000	0.000000
0.000000	3.864016	2.707714	-0.000000	0.000000	-0.000000
2.795505	3.864016	0.000000	-0.000000	0.000000	0.000000
2.659869	1.932008	4.933751	0.000000	-0.026823	0.000000
2.931141	5.796024	0.481677	0.000000	0.026823	0.000000
0.135636	5.796024	2.226037	-0.000000	0.026823	0.000000
5.455373	1.932008	3.189392	-0.000000	-0.026823	0.000000
4.440201	0.366692	1.112822	0.018286	-0.114902	-0.015637
1.150808	7.361340	4.302607	-0.018286	0.114902	0.015637
3.946313	7.361340	3.820536	-0.018286	0.114902	-0.015637
1.644697	0.366692	1.594893	0.018286	-0.114902	0.015637
1.150808	4.230709	4.302607	0.018286	0.114902	-0.015637
4.440201	3.497324	1.112822	-0.018286	-0.114902	0.015637
1.644697	3.497324	1.594893	-0.018286	-0.114902	-0.015637
3.946313	4.230709	3.820536	0.018286	0.114902	0.015637

--

B_{1g}(2); Nd(y), out-of-phase

50 f = 4.715142 THz 29.626113 2PiTHz 157.280215 cm-1 19.500268 meV

X	Y	Z	dx	dy	dz
0.303633	1.932008	0.073534	-0.494733	-0.000000	0.042296

5.287376	5.796024	5.341894	0.494733	0.000000	-0.042296
2.491871	5.796024	2.781249	-0.494733	-0.000000	-0.042296
3.099138	1.932008	2.634180	0.494733	0.000000	0.042296
0.000000	0.000000	2.707714	-0.000000	-0.000000	-0.000000
2.795505	0.000000	0.000000	-0.000000	0.000000	-0.000000
0.000000	3.864016	2.707714	0.000000	-0.000000	0.000000
2.795505	3.864016	0.000000	-0.000000	0.000000	0.000000
2.659869	1.932008	4.933751	-0.040035	0.000000	0.007103
2.931141	5.796024	0.481677	0.040035	0.000000	-0.007103
0.135636	5.796024	2.226037	-0.040035	0.000000	-0.007103
5.455373	1.932008	3.189392	0.040035	-0.000000	0.007103
4.440201	0.366692	1.112822	-0.004099	0.025002	0.016015
1.150808	7.361340	4.302607	0.004099	-0.025002	-0.016015
3.946313	7.361340	3.820536	-0.004099	0.025002	-0.016015
1.644697	0.366692	1.594893	0.004099	-0.025002	0.016015
1.150808	4.230709	4.302607	0.004099	0.025002	-0.016015
4.440201	3.497324	1.112822	-0.004099	-0.025002	0.016015
1.644697	3.497324	1.594893	0.004099	0.025002	0.016015
3.946313	4.230709	3.820536	-0.004099	-0.025002	-0.016015

--

A_g(2); Nd(x), out-of-phase

51 f = 4.239935 THz 26.640300 2PiTHz 141.429020 cm-1 17.534970 meV

X	Y	Z	dx	dy	dz
0.303633	1.932008	0.073534	-0.018336	-0.000000	0.468851
5.287376	5.796024	5.341894	0.018336	0.000000	-0.468851
2.491871	5.796024	2.781249	0.018336	-0.000000	0.468851
3.099138	1.932008	2.634180	-0.018336	0.000000	-0.468851
0.000000	0.000000	2.707714	0.000000	-0.000000	-0.000000
2.795505	0.000000	0.000000	0.000000	0.000000	-0.000000

0.000000	3.864016	2.707714	-0.000000	-0.000000	0.000000
2.795505	3.864016	0.000000	0.000000	-0.000000	0.000000
2.659869	1.932008	4.933751	-0.005227	0.000000	-0.072957
2.931141	5.796024	0.481677	0.005227	-0.000000	0.072957
0.135636	5.796024	2.226037	0.005227	-0.000000	-0.072957
5.455373	1.932008	3.189392	-0.005227	-0.000000	0.072957
4.440201	0.366692	1.112822	-0.069298	0.056194	0.065470
1.150808	7.361340	4.302607	0.069298	-0.056194	-0.065470
3.946313	7.361340	3.820536	0.069298	-0.056194	0.065470
1.644697	0.366692	1.594893	-0.069298	0.056194	-0.065470
1.150808	4.230709	4.302607	0.069298	0.056194	-0.065470
4.440201	3.497324	1.112822	-0.069298	-0.056194	0.065470
1.644697	3.497324	1.594893	-0.069298	-0.056194	-0.065470
3.946313	4.230709	3.820536	0.069298	0.056194	0.065470

--

$B_{2g}(1)$; Nd(z) out-of-phase in x-y, z

52 f = 4.223338 THz 26.536015 2PiTHz 140.875386 cm-1 17.466328 meV

X	Y	Z	dx	dy	dz
0.303633	1.932008	0.073534	-0.000000	0.494394	0.000000
5.287376	5.796024	5.341894	0.000000	-0.494394	-0.000000
2.491871	5.796024	2.781249	0.000000	0.494394	0.000000
3.099138	1.932008	2.634180	0.000000	-0.494394	-0.000000
0.000000	0.000000	2.707714	0.000000	0.000000	0.000000
2.795505	0.000000	0.000000	0.000000	0.000000	0.000000
0.000000	3.864016	2.707714	-0.000000	0.000000	-0.000000
2.795505	3.864016	0.000000	-0.000000	0.000000	-0.000000
2.659869	1.932008	4.933751	-0.000000	-0.005332	-0.000000
2.931141	5.796024	0.481677	0.000000	0.005332	0.000000
0.135636	5.796024	2.226037	0.000000	-0.005332	-0.000000

5.455373	1.932008	3.189392	-0.000000	0.005332	0.000000
4.440201	0.366692	1.112822	-0.004909	0.051579	0.009408
1.150808	7.361340	4.302607	0.004909	-0.051579	-0.009408
3.946313	7.361340	3.820536	-0.004909	0.051579	-0.009408
1.644697	0.366692	1.594893	0.004909	-0.051579	0.009408
1.150808	4.230709	4.302607	-0.004909	-0.051579	0.009408
4.440201	3.497324	1.112822	0.004909	0.051579	-0.009408
1.644697	3.497324	1.594893	-0.004909	-0.051579	-0.009408
3.946313	4.230709	3.820536	0.004909	0.051579	0.009408

--

B_{3u}(1); Nd(x) , in-phase in z, out-of-phase in y, Fe(y)

53 f = 3.447834 THz 21.663379 2PiTHz 115.007355 cm-1 14.259100 meV

X	Y	Z	dx	dy	dz
0.303633	1.932008	0.073534	-0.446834	-0.000000	-0.016125
5.287376	5.796024	5.341894	-0.446834	0.000000	-0.016125
2.491871	5.796024	2.781249	0.446834	0.000000	-0.016125
3.099138	1.932008	2.634180	0.446834	0.000000	-0.016125
0.000000	0.000000	2.707714	0.202527	-0.040709	0.003545
2.795505	0.000000	0.000000	-0.202527	0.040709	0.003545
0.000000	3.864016	2.707714	0.202527	0.040709	0.003545
2.795505	3.864016	0.000000	-0.202527	-0.040709	0.003545
2.659869	1.932008	4.933751	-0.048559	0.000000	-0.010553
2.931141	5.796024	0.481677	-0.048559	0.000000	-0.010553
0.135636	5.796024	2.226037	0.048559	-0.000000	-0.010553
5.455373	1.932008	3.189392	0.048559	-0.000000	-0.010553
4.440201	0.366692	1.112822	-0.018923	-0.037718	0.026091
1.150808	7.361340	4.302607	-0.018923	-0.037718	0.026091
3.946313	7.361340	3.820536	0.018923	0.037718	0.026091
1.644697	0.366692	1.594893	0.018923	0.037718	0.026091

1.150808	4.230709	4.302607	-0.018923	0.037718	0.026091
4.440201	3.497324	1.112822	-0.018923	0.037718	0.026091
1.644697	3.497324	1.594893	0.018923	-0.037718	0.026091
3.946313	4.230709	3.820536	0.018923	-0.037718	0.026091

--

B_{2u}(1); Nd(x) in-phase in z, out-of-phase in x-y; Fe(x)

54 f = 3.306047 THz 20.772509 2PiTHz 110.277869 cm-1 13.672718 meV

X	Y	Z	dx	dy	dz
0.303633	1.932008	0.073534	-0.041309	0.000000	-0.445122
5.287376	5.796024	5.341894	-0.041309	-0.000000	-0.445122
2.491871	5.796024	2.781249	-0.041309	0.000000	0.445122
3.099138	1.932008	2.634180	-0.041309	0.000000	0.445122
0.000000	0.000000	2.707714	0.030105	-0.010060	-0.204385
2.795505	0.000000	0.000000	0.030105	-0.010060	0.204385
0.000000	3.864016	2.707714	0.030105	0.010060	-0.204385
2.795505	3.864016	0.000000	0.030105	0.010060	0.204385
2.659869	1.932008	4.933751	0.006744	0.000000	0.059861
2.931141	5.796024	0.481677	0.006744	0.000000	0.059861
0.135636	5.796024	2.226037	0.006744	-0.000000	-0.059861
5.455373	1.932008	3.189392	0.006744	0.000000	-0.059861
4.440201	0.366692	1.112822	0.030302	0.019203	-0.024258
1.150808	7.361340	4.302607	0.030302	0.019203	-0.024258
3.946313	7.361340	3.820536	0.030302	0.019203	0.024258
1.644697	0.366692	1.594893	0.030302	0.019203	0.024258
1.150808	4.230709	4.302607	0.030302	-0.019203	-0.024258
4.440201	3.497324	1.112822	0.030302	-0.019203	-0.024258
1.644697	3.497324	1.594893	0.030302	-0.019203	0.024258
3.946313	4.230709	3.820536	0.030302	-0.019203	0.024258

--

$B_{1g}(1)$; Nd(x), in-phase in x-y, out-of-phase in z

55 f = 3.298030 THz 20.722132 2PiTHz 110.010429 cm-1 13.639560 meV

X	Y	Z	dx	dy	dz
0.303633	1.932008	0.073534	-0.044000	-0.000000	-0.491268
5.287376	5.796024	5.341894	0.044000	-0.000000	0.491268
2.491871	5.796024	2.781249	-0.044000	0.000000	0.491268
3.099138	1.932008	2.634180	0.044000	0.000000	-0.491268
0.000000	0.000000	2.707714	-0.000000	-0.000000	-0.000000
2.795505	0.000000	0.000000	0.000000	-0.000000	0.000000
0.000000	3.864016	2.707714	-0.000000	-0.000000	-0.000000
2.795505	3.864016	0.000000	0.000000	0.000000	0.000000
2.659869	1.932008	4.933751	0.000432	0.000000	-0.070912
2.931141	5.796024	0.481677	-0.000432	0.000000	0.070912
0.135636	5.796024	2.226037	0.000432	-0.000000	0.070912
5.455373	1.932008	3.189392	-0.000432	0.000000	-0.070912
4.440201	0.366692	1.112822	0.015513	0.008051	-0.023237
1.150808	7.361340	4.302607	-0.015513	-0.008051	0.023237
3.946313	7.361340	3.820536	0.015513	0.008051	0.023237
1.644697	0.366692	1.594893	-0.015513	-0.008051	-0.023237
1.150808	4.230709	4.302607	-0.015513	0.008051	0.023237
4.440201	3.497324	1.112822	0.015513	-0.008051	-0.023237
1.644697	3.497324	1.594893	-0.015513	0.008051	-0.023237
3.946313	4.230709	3.820536	0.015513	-0.008051	0.023237

--

$A_g(1)$; R(y), in-phase in x-y, out-of-phase in z

56 f = 3.135370 THz 19.700113 2PiTHz 104.584696 cm-1 12.966854 meV

X	Y	Z	dx	dy	dz
0.303633	1.932008	0.073534	0.480217	0.000000	0.053685
5.287376	5.796024	5.341894	-0.480217	0.000000	-0.053685

2.491871	5.796024	2.781249	-0.480217	0.000000	0.053685
3.099138	1.932008	2.634180	0.480217	-0.000000	-0.053685
0.000000	0.000000	2.707714	-0.000000	0.000000	0.000000
2.795505	0.000000	0.000000	0.000000	-0.000000	-0.000000
0.000000	3.864016	2.707714	-0.000000	-0.000000	0.000000
2.795505	3.864016	0.000000	0.000000	0.000000	-0.000000
2.659869	1.932008	4.933751	0.050273	-0.000000	-0.005259
2.931141	5.796024	0.481677	-0.050273	0.000000	0.005259
0.135636	5.796024	2.226037	-0.050273	-0.000000	-0.005259
5.455373	1.932008	3.189392	0.050273	0.000000	0.005259
4.440201	0.366692	1.112822	0.065009	-0.005780	-0.052133
1.150808	7.361340	4.302607	-0.065009	0.005780	0.052133
3.946313	7.361340	3.820536	-0.065009	0.005780	-0.052133
1.644697	0.366692	1.594893	0.065009	-0.005780	0.052133
1.150808	4.230709	4.302607	-0.065009	-0.005780	0.052133
4.440201	3.497324	1.112822	0.065009	0.005780	-0.052133
1.644697	3.497324	1.594893	0.065009	0.005780	0.052133
3.946313	4.230709	3.820536	-0.065009	-0.005780	-0.052133

--

A_u(1) silent mode

57 f = 2.678165 THz 16.827404 2PiTHz 89.333949 cm-1 11.076002 meV

X	Y	Z	dx	dy	dz
0.303633	1.932008	0.073534	0.000000	-0.463544	0.000000
5.287376	5.796024	5.341894	-0.000000	-0.463544	-0.000000
2.491871	5.796024	2.781249	-0.000000	0.463544	-0.000000
3.099138	1.932008	2.634180	-0.000000	0.463544	0.000000
0.000000	0.000000	2.707714	-0.051986	0.011989	0.109304
2.795505	0.000000	0.000000	0.051986	-0.011989	0.109304
0.000000	3.864016	2.707714	0.051986	0.011989	-0.109304

2.795505	3.864016	0.000000	-0.051986	-0.011989	-0.109304
2.659869	1.932008	4.933751	0.000000	-0.020751	-0.000000
2.931141	5.796024	0.481677	-0.000000	-0.020751	-0.000000
0.135636	5.796024	2.226037	0.000000	0.020751	-0.000000
5.455373	1.932008	3.189392	-0.000000	0.020751	-0.000000
4.440201	0.366692	1.112822	-0.035626	-0.004868	0.093051
1.150808	7.361340	4.302607	-0.035626	-0.004868	0.093051
3.946313	7.361340	3.820536	0.035626	0.004868	0.093051
1.644697	0.366692	1.594893	0.035626	0.004868	0.093051
1.150808	4.230709	4.302607	0.035626	-0.004868	-0.093051
4.440201	3.497324	1.112822	0.035626	-0.004868	-0.093051
1.644697	3.497324	1.594893	-0.035626	0.004868	-0.093051
3.946313	4.230709	3.820536	-0.035626	0.004868	-0.093051

--

X -translation mode

58 f/i= 0.016352 THz 0.102745 2PiTHz 0.545457 cm-1 0.067628 meV

X	Y	Z	dx	dy	dz
0.303633	1.932008	0.073534	0.000014	-0.000000	-0.381567
5.287376	5.796024	5.341894	0.000014	-0.000000	-0.381567
2.491871	5.796024	2.781249	-0.000014	-0.000000	-0.381567
3.099138	1.932008	2.634180	-0.000014	-0.000000	-0.381567
0.000000	0.000000	2.707714	-0.000125	-0.000104	-0.236343
2.795505	0.000000	0.000000	0.000125	0.000104	-0.236343
0.000000	3.864016	2.707714	-0.000125	0.000104	-0.236343
2.795505	3.864016	0.000000	0.000125	-0.000104	-0.236343
2.659869	1.932008	4.933751	-0.000041	-0.000000	-0.127262
2.931141	5.796024	0.481677	-0.000041	-0.000000	-0.127262
0.135636	5.796024	2.226037	0.000041	-0.000000	-0.127262
5.455373	1.932008	3.189392	0.000041	-0.000000	-0.127262

4.440201	0.366692	1.112822	0.000118	-0.000113	-0.127186
1.150808	7.361340	4.302607	0.000118	-0.000113	-0.127186
3.946313	7.361340	3.820536	-0.000118	0.000113	-0.127186
1.644697	0.366692	1.594893	-0.000118	0.000113	-0.127186
1.150808	4.230709	4.302607	0.000118	0.000113	-0.127186
4.440201	3.497324	1.112822	0.000118	0.000113	-0.127186
1.644697	3.497324	1.594893	-0.000118	-0.000113	-0.127186
3.946313	4.230709	3.820536	-0.000118	-0.000113	-0.127186

--

Z -translation mode

59 f/i= 0.016858 THz 0.105920 2PiTHz 0.562314 cm-1 0.069718 meV

X	Y	Z	dx	dy	dz
0.303633	1.932008	0.073534	0.000000	0.381596	-0.000000
5.287376	5.796024	5.341894	0.000000	0.381596	-0.000000
2.491871	5.796024	2.781249	0.000000	0.381596	-0.000000
3.099138	1.932008	2.634180	0.000000	0.381596	-0.000000
0.000000	0.000000	2.707714	-0.000330	0.236317	-0.000183
2.795505	0.000000	0.000000	-0.000330	0.236317	0.000183
0.000000	3.864016	2.707714	0.000330	0.236317	0.000183
2.795505	3.864016	0.000000	0.000330	0.236317	-0.000183
2.659869	1.932008	4.933751	0.000000	0.126945	-0.000000
2.931141	5.796024	0.481677	0.000000	0.126945	-0.000000
0.135636	5.796024	2.226037	0.000000	0.126945	-0.000000
5.455373	1.932008	3.189392	0.000000	0.126945	-0.000000
4.440201	0.366692	1.112822	-0.000134	0.127326	0.000068
1.150808	7.361340	4.302607	-0.000134	0.127326	0.000068
3.946313	7.361340	3.820536	-0.000134	0.127326	-0.000068
1.644697	0.366692	1.594893	-0.000134	0.127326	-0.000068
1.150808	4.230709	4.302607	0.000134	0.127326	-0.000068

4.440201	3.497324	1.112822	0.000134	0.127326	-0.000068
1.644697	3.497324	1.594893	0.000134	0.127326	0.000068
3.946313	4.230709	3.820536	0.000134	0.127326	0.000068

--

Y -translation mode

60 f/i= 0.022685 THz 0.142531 2PiTHz 0.756675 cm-1 0.093816 meV

X	Y	Z	dx	dy	dz
0.303633	1.932008	0.073534	0.381792	-0.000000	-0.000123
5.287376	5.796024	5.341894	0.381792	-0.000000	-0.000123
2.491871	5.796024	2.781249	0.381792	-0.000000	0.000123
3.099138	1.932008	2.634180	0.381792	-0.000000	0.000123
0.000000	0.000000	2.707714	0.235895	-0.000164	-0.000169
2.795505	0.000000	0.000000	0.235895	-0.000164	0.000169
0.000000	3.864016	2.707714	0.235895	0.000164	-0.000169
2.795505	3.864016	0.000000	0.235895	0.000164	0.000169
2.659869	1.932008	4.933751	0.127370	-0.000000	0.000053
2.931141	5.796024	0.481677	0.127370	-0.000000	0.000053
0.135636	5.796024	2.226037	0.127370	-0.000000	-0.000053
5.455373	1.932008	3.189392	0.127370	-0.000000	-0.000053
4.440201	0.366692	1.112822	0.127211	0.000002	-0.000146
1.150808	7.361340	4.302607	0.127211	0.000002	-0.000146
3.946313	7.361340	3.820536	0.127211	0.000002	0.000146
1.644697	0.366692	1.594893	0.127211	0.000002	0.000146
1.150808	4.230709	4.302607	0.127211	-0.000002	-0.000146
4.440201	3.497324	1.112822	0.127211	-0.000002	-0.000146
1.644697	3.497324	1.594893	0.127211	-0.000002	0.000146
3.946313	4.230709	3.820536	0.127211	-0.000002	0.000146
